# Relationship between quadriceps femoris muscle architecture and muscle strength and physical function in older adults with heart failure with preserved ejection fraction

**DOI:** 10.1038/s41598-022-26064-7

**Published:** 2022-12-15

**Authors:** Iván J. Fuentes-Abolafio, M. Rosa Bernal-López, Ricardo Gómez-Huelgas, Michele Ricci, Antonio I. Cuesta-Vargas, Luis M. Pérez-Belmonte

**Affiliations:** 1 Grupo de Investigación Clinimetría CTS-631, Departamento de Fisioterapia, Facultad de Ciencias de la Salud, Universidad de Málaga, C/Arquitecto Peñalosa, 3, 29071 Málaga, España; 2 Instituto de Investigación Biomédica de Málaga y Plataforma en Nanomedicina-IBIMA Plataforma BIONAND, Málaga, España; 3 Departamento de Medicina Interna, Hospital Regional Universitario de Málaga, Málaga, España; 4 CIBER Fisio-Patología de la Obesidad y la Nutrición, Instituto de Salud Carlos III, Madrid, España; 5 School of Clinical Sciences, Faculty of Health at the Queensland University of Technology, Brisbane, QLD Australia; 6 Unidad de Neurofisiología Cognitiva, Centro de Investigaciones Médico Sanitarias (CIMES), Universidad de Málaga (UMA), Campus de Excelencia Internacional (CEI) Andalucía Tech, Málaga, España; 7 Centro de Investigación Biomédica en Red Enfermedades Cardiovasculares (CIBERCV), Instituto de Salud Carlos III, Madrid, España

**Keywords:** Biomarkers, Cardiology

## Abstract

Heart failure (HF)-related factors potentially lead to sarcopenia. Ultrasound (US) assessment has all the advantages of being used in clinical practice to assess muscle architecture. This study aimed to assess the relationship between the quadriceps femoris (QF) muscle architecture with the gender, age, body mass index (BMI), muscle strength and physical function in older adults with HF with preserved ejection fraction (HFpEF) as well as to assess the difference in these relationships between the two genders. Patients 70 years and older with HFpEF were included. The gender, age and BMI were collected. The QF muscle thickness, the QF muscle echo-intensity, the subcutaneous fat tissue thickness (FT) and the subcutaneous fat tissue echo-intensity were assessed by the US. The six-minute walk test, the short physical performance battery (SPPB), the timed up and go test (TUG), and the gait speed test (UGS) were used to assess physical function. The five-repetitions sit-to-stand test (5-STS) was performed to assess muscle strength. Bivariant Pearson correlations and subsequent multivariate linear regression analysis were conducted. Seventy older adults with HFpEF [81.00 (5.97) years] were recruited. The FT showed a correlation between poor and moderate muscle strength and physical function in women with HFpEF. The FT explained 24.5% of the 5-STS variance, 32.4% of the SPPB variance, 31.5% of the TUG variance, 28.6% of the UGS variance, and 21.4% of the FGS variance in women. The US assessment could allow clinicians to assess muscle architecture biomarkers related to muscle strength and physical function in older adults with HFpEF.

*Trial registration* NCT03909919. April 10, 2019. Retrospectively registered.

## Introduction

Heart Failure (HF) is a chronic and clinical syndrome with symptoms and/or signs caused by a structural or functional cardiac abnormality^1^. The HF worldwide prevalence ranges from 1 to 3%^2^. Patients with heart failure with a preserved ejection fraction (HFpEF) account for more than 50% of all patients with HF^1,2^. Comorbidity is common and more severe in patients with HFpEF than in patients with heart failure with reduced ejection fraction (HFrEF)^1^. Sarcopenia is one of the most important comorbidities in patients with HF^1^. HF-related factors potentially lead to sarcopenia, like hormonal changes, physical inactivity, oxidative stress or inflammation^3^. Sarcopenia has been associated with a worse prognosis and reduced functional aerobic capacity and quality of life in patients with HFpEF^4,5^.

Sarcopenia is defined as a muscle disease characterised by low muscle strength and low muscle quantity and quality^6^. Moreover, sarcopenia is considered as severe if the physical function is poor^6^. The grip strength test or the chair stand test was suggested to assess muscle strength, while the short physical performance battery (SPPB), the timed up and go test (TUG) or the gait speed test were recommended to assess physical function^6^. The skeletal muscle mass (SMM) or appendicular skeletal muscle mass (ASM) were suggested to delimit the muscle quantity^6^. Magnetic resonance imaging (MRI) and computed tomography (CT) are gold standards for noninvasive muscle quantity and quality assessment^7,8^. However, these tools are not commonly used in primary care because of high equipment costs and the lack of portability^7,8^. Ultrasound (US) assessment has shown good validity in estimating muscle mass compared to MRI and CT^9^. US is a reliable and valid tool to assess the muscle quantity of pennate muscles in older adults, such as the quadriceps femoris (QF) muscle^9^. The US can assess muscle quality by analysing the echogenicity or echo-intensity^10^. Thus, the US allows assessing muscle quantity and quality with high resolution within a relatively short period^10,11^. Moreover, the US is a portable, cheap, simple, easy-to-use, and widely available clinical practice technique that can be performed bedside and enables the physician to visualise a wide range of components of the muscle architecture and the fat tissue^11,12^. In this way, the US has sufficient potential to be used in clinical practice to screen for sarcopenia and assess body composition or muscle architecture^12^.

The QF is the most investigated muscle because it is easy to measure by the US; it is a good predictor of whole-body skeletal muscle mass and can be linked directly to physical function measures^9,13^. Kawai et al.^13^ showed that four structural QF biomarkers assessed by the US, muscle and subcutaneous fat tissue thickness and echo-intensity, are associated with muscle strength, physical function, and sarcopenia in community-dwelling older adults. The association between these US biomarkers and muscle strength or physical function has not been studied in older adults with HFpEF. Therefore, the objectives of the present study were to assess the relationship between the QF muscle and subcutaneous fat tissue thickness and echo-intensity with the gender, age, body mass index (BMI), muscle strength and physical function in older adults with HFpEF, and to assess the difference in these relationships between the two genders.

## Methods

### Design and participants

A cross-sectional study was carried out. Seventy older adults with HFpEF were recruited as volunteers between April 2019 and March 2020 from the Heart Failure Unit of the Internal Medicine Department at the Regional University Hospital of Malaga (Spain). Inclusion criteria^1^: patients with HFpEF older than 70 years diagnosed according to the consensus statement of the European Society of Cardiology^14^. Exclusion criteria: (1) older adults with HFpEF with a New York Heart Association (NYHA) class = 4; (2) older adults hospitalised 3 months ago or less; (3) older adults with a score on the Mini-Mental State Examination (MMSE) < 24; (4) older adults who were not able to stand up from the chair at least five times or who were not able to walk.

### Outcomes

#### US biomarkers

The US analysed the right QF muscle and the subcutaneous fat tissue. The following variables were analysed:

- Thickness: Thickness refers to the width of the QF muscle or subcutaneous fat tissue. It was calculated using a perpendicular line to the horizontal axis from the midpoint of the femur to standardise the measurement. This line was placed between the femur and the superior fascia to assess the QF muscle thickness (MT). This measurement showed a high intra-rater and inter-rater reliability, with an intraclass correlation coefficient (ICC) of 0.98 and 0.96, respectively^15^. This measurement also showed an absolute error between days of 0.017 cm^16^. The perpendicular line was placed between the superior fascia and the skin to assess the fat thickness (FT). The values were expressed in cm.
- Echo-intensity: Echo-intensity is calculated from the selected range of interest as the average result of the histogram of the 8-bit grayscale, so the echo-intensity represents the mean pixel intensity. The resultant histogram analysed all the image pixels from 0, black, to 255, white. This outcome has no unit of measurement.

The combination of these variables (thickness and echo-intensity) in contraction (con) and non-contraction (non-con) situations and in different tissues (QF muscle and subcutaneous fat tissue) allowed obtaining twelve variables: non-con MT, non-con muscle echo-intensity (MEI), non-con FT, non-con fat echo-intensity (FEI), con MT, con MEI, con FT, con FEI, the difference between con and non-con MT and FT, and the difference between con and non-con MEI and FEI.

The US image was taken 15 cm from the upper edge of the patella, where transverse images were obtained with a B-mode ultrasound device (The Esaote MyLab One; Esaote, Genova, Italy) equipped with a linear array transducer 5 cm long. The transducer was placed perpendicular to the axis of the limb and transversely to the direction of the fibres. Older adults were seated in a chair with their hip and knee at 90° of flexion (Supplementary Appendix B). The evaluator was placed in front of the patient, holding the transducer with one hand and the participant's leg with the other one. Before performing the US measurements, the older adults rested for 5 min to avoid bias in measuring muscle thickness and echo-intensity. To adequately capture a static image in a contraction state, the participant performed a manually resisted voluntary isometric contraction of 5 s by the physician. The parameters used to acquire US images included the B-mode, a frequency of 10 MHz, 4 cm deep, and 42% of the gain. Coupling gel was abundantly applied to minimise distortion generated by underlying tissues. Shaving was not needed.

### Secondary outcomes

Clinical-epidemiological: age, gender, NYHA class, comorbidities, echocardiographic outcomes, blood and urinary biomarkers, number of drugs that the patient takes each day and the most prescribed drugs, history of smoking and history of alcohol, marital status, academic degree and the number of falls in the last year.

Anthropometric data: weight, height, and BMI.

### Muscle strength

Five-Repetitions Sit-to-Stand (5-STS): older adults should stand up and sit down five times as quickly as possible without using their hands to push up from the chair. The back of the chair was stabilised against a wall to ensure safety and stability. The time taken to perform the five repetitions was measured using a stopwatch^17^.

### Physical function

Short Physical Performance Battery (SPPB) is formed by three tests: balance (feet together, semi tandem and tandem for 10 s each), 4 m (m) gait speed and the 5-STS. Each test is scored from 0 (worst performance) to 4 (best performance). A score of 0 is assigned to those older adults who do not complete the test. Scores from 1 to 4 are based on older adults' time performing each test. The total score for the whole battery is the addition of the 3 tests and ranges from 0 to 12 points^18^.

Timed Up and Go Test (TUG). In this test, the patient started sitting in a chair. When the physician indicated the beginning of the test, they stood up from the chair and walked 3 m at a pace as quickly, comfortably and safely as possible until to reach a line on the floor. Then, older adults turned, returned to the chair, walked and sat again^19^. Patients could use their hands to stand up from the chair. The score was the seconds taken to complete the test, measured by a stopwatch.

Six Minute Walking Test (6MWT*)* was carried out in a closed corridor longer than 30 m. Two marks were placed on the ground at a distance of 30 m, and older adults walked from one end to the other for 6 min. Older adults were instructed to walk as quickly as possible. The distance older adults walked for 6 min was recorded^20^.

Gait speed test. Older adults should walk 4 m, starting from a standing position. The test was performed twice: at their usual pace or gait speed (UGS) and the other at a fast pace or gait speed (FGS). The time taken to perform the 4 m was measured using a stopwatch, and the gait speed was calculated as m/sec^21^.

### Self-reported

Abbreviated Comprehensive Geriatric Assessment (aCGA). aCGA assesses functional, emotional and cognitive components, and it is a short version of a comprehensive geriatric assessment with greater reliability^22^.

### Sample size

The sample size was calculated using the software G Power 3.1.9.2 (University of Düsseldorf, Germany) and following the alternative hypothesis: to detect a moderate bivariate correlation (r = 0.3)^13^ between the US biomarkers and the muscle strength or the physical function, considering a significance level of 0.05 (error α < 5%), and statistical power of 0.8 (80%), a sample consisting of 67 older adults with HFpEF would be needed.

### US data processing and analysis

US images were exported in BMP format with a specific size of 800 × 652 pixels and 100 dpi. MATLAB software (Version R2018b, MathWorks, Natick, USA) was used to perform the image processing and analysis. An own MATLAB code was created specifically for this project. In this code, the researcher had to record a reference line of 1 cm, which formed the width of the range of interest. The research could rely on the line that shows the centimetres of the depth of the US image to record the reference line of 1 cm. Then, the researcher could select a range of interest with a width of 1 cm and a height from the femur to the superficial layer of the skin (Supplementary Appendix C). This type of assessment showed a high test–retest reliability score (ICC = 0.963) with an average coefficient of variation of 4.2%^23^. Once the range of interest is selected, the code converts the image to grayscale. The following three points were taken as references: the superior limit of the femur bone, the inferior limit of the skin, and the superior limit of the fascia between QF muscle and subcutaneous fat tissue. The same evaluator performed the MATLAB analysis of all images to reduce inter-rater variability.

### Statistical analysis

An absolute frequency and a percentage were used to describe qualitative measures. Quantitative measures were reported using the mean and the standard deviation (SD). Distribution and normality were determined by one-sample Kolmogorov–Smirnov test (significance < 0.05). The Student's t-test (t-test) and the Chi-square test were used to compare the outcomes between men and women. Levene's test assessed the variance heterogeneity (significance < 0.15). The Pearson Correlation Coefficient (r) was used to assess the possible bivariate correlations between the US biomarkers, age, BMI, muscle strength and physical function, stratified by gender. Spearman's rho (ρ) was used to assess the correlations between the US biomarkers and self-reported outcomes (NYHA, Katz and Lawnton & Brody questionnaires). Partial correlation coefficients were calculated, stratified by gender while controlling for BMI. Bivariate correlations were classified into three categories: poor (r ≤ 0.49), moderate (0.50 ≤ r ≤ 0.74) and strong (r ≥ 0.75). Multivariate linear regression analyses were performed to assess the relationship between the US biomarkers and muscle strength and physical function. Only the US biomarkers that showed the most significant bivariate correlation with muscle strength or physical function were included in the model, adjusted by age and BMI and stratified by gender. The contribution of the exposures to the model's predictability was assessed by the coefficient of determination (R2). A confirmatory factor analysis with principal component analysis (PCA) was employed to determine what US biomarkers could construct a classification system with standard scores. Kaiser–Meyer–Oklin (KMO) values and Bartlett's test of sphericity were analysed to assess the model adequacy. The US biomarkers determined by the PCA were used to identify homogeneous subtypes in an exploratory hierarchical cluster. The Ward's linkage method to form clusters at each stage squared euclidean distances included in the proximities matrix, and the standardisation of the US biomarkers (z-scores) were used. Agglomeration coefficients were examined and plotted to identify the best cluster solution representing the data. The per-cent changes between adjacent cluster solutions and plot characteristics were considered. The dendrogram, which represents the relationships of similarity among the group of clusters, was also visually inspected to decide the number of clusters. One-way analysis of variance (ANOVA) was employed to examine the significant differences in all outcomes among the classification types. When the variances were homogeneity, the post hoc Bonferroni test was used, while the post hoc Games-Howell test was used when variances were heterogeneous. A *p*-value of *p* < 0.05 was considered to be statistically significant. All statistical analyses were conducted using the Statistical Package for the Social Sciences (SPSS) 22.0 for Windows.

### Ethics and consent

The study was registered on the ClinicalTrial.gov database as NCT03909919. Ethical approval was obtained from the Provincial Ethics Committee of Malaga, Spain (26032020). The study was carried out following the Helsinki Declaration and was implemented and reported according to the Strengthening the Reporting of Observational Studies in Epidemiology Statement (STROBE) (Supplementary Appendix A). Moreover, all participants in this study signed an informed consent form prior to enrolment.

## Results

Seventy six older adults with HFpEF were voluntarily recruited, but data for six older adults were lost when outcomes were collected. Thus, data of seventy older adults with HFpEF were included in the present study. Height and body weight were significantly greater in men than in women. However, there was no difference in BMI between gender. Non-con MEI and con MEI were greater in men than in women, whereas non-con FT, non-con FEI, con FT and con FEI were significantly greater in women than in men. Women also showed a worse physical functional performance (SPPB, TUG and 6MWT) and slower UGS and FGS than men. There was no difference in self-reported outcomes and age between gender (Supplementary Appendix D). Other clinical-epidemiological variables and blood are shown in Supplementary Appendix E. In summary, the mean LVEF was 60.50%, and most of the older adults with HFpEF were overweight (41.40%) or obese (35.70%). Forty older adults with HFpEF (57.10%) had fallen last year. In addition, older adults with HFpEF had a mean of 8.31 comorbidities and were taking a mean of 10.16 drugs per day. The most frequent comorbidities were hypertension (97.10%), dyslipidemia (85.70%), valve disease (65.70%) and chronic kidney disease (64.30%). In addition, older adults with HFpEF showed a left atrial dimension of 42.23 mm, a left ventricular end-systolic dimension of 29.48 mm and a left ventricular end-diastolic dimension of 47.79 mm. The most prescribed drugs were loop diuretics (85.71%), beta-blockers (75.70%) and angiotensin II receptor antagonists (62.90%).

Non-con FT and con FT significantly correlated with women's muscle strength and physical function. Non-con and con FT also significantly correlated with BMI in women and men (Table 1). Con MT was inversely correlated with age while con MEI was directly correlated with age in women. That is, the older the women with HFpEF, the lower the MT and worse muscle quality. When adjusted for BMI, non-con FT and con FT showed a significant correlation with muscle strength and physical function in women, except with the 6MWT (Supplementary Appendix F). Non-con and con FT explained 24.5% of the 5-STS variance, 32.4% of the SPPB variance, 31.5% of the TUG variance, 2865% of the UGS variance, and 21.4% of the FGS variance in women (Table 2). However, these US biomarkers did not reach the required value to explain the model (Table 3).

**Table 1:** Bivariate correlations (r, ρ) between the US biomarkers and age, BMI, self-reported questionnaires, muscle strength and physical functional performance, stratified by gender.

	Age	BMI	NYHA	Katz Index	Lawnton & Brody	5-STS	SPPB	TUG	6MWT	UGS	FGS
**Men (n = 30)**											
Non-con MT	− .151	.450*	− .092	− .075	.305	.192	− .120	.379*	− .497*	− .297	− .152
Non-con MEI	− .348	− .268	.089	− .042	.131	.123	− .222	.082	− .008	− .183	.071
Non-con FT	.280	.393*	− .012	− .092	.122	− .243	.184	− .177	.102	.135	.207
Non-con FEI	.256	.095	.036	− .058	− .039	− .233	.136	− .310	.373*	.219	.204
Con MT	− .164	.511*	− .212	.055	.300	.090	− .040	.305	− .317	− .171	.039
Con MEI	− .181	− .484*	.167	− .072	− .129	.057	− .060	.008	− .022	− .124	.115
Con FT	.205	.432*	.076	− .217	.196	− .194	.143	− .141	.098	.144	.168
Con FEI	.196	.031	.140	− .156	− .160	− .072	.005	− .179	.195	.099	.031
MT Difference	− .061	.232	− .276	.261	.070	− .170	.140	− .066	.263	.196	.366*
MEI Difference	.240	− .219	.108	.098	− .459*	− .093	.218	− .098	− .015	.092	.043
FT Difference	− .513*	.051	.260	− .333	.288	.358	− .293	.258	− .061	− .005	− .288
FEI Difference	− .126	− .116	.220	− .183	− .130	.291	− .232	.250	− .335	− .223	− .308
**Women (n = 40)**											
Non-con MT	− .291	.233	.395*	.202	− .037	.283	− .242	.190	− .147	− .113	− .226
Non-con MEI	.250	− .406*	.051	− .140	.104	− .160	.224	− .224	.074	.188	.175
Non-con FT	− .143	.494**	− .056	.218	− .280	.469*	− .555**	.539**	− .365*	− .503**	− .445*
Non-con FEI	.187	.122	− .323*	.112	− .266	.190	− .268	.244	− .288	− .354*	− .211
Con MT	− .314*	.210	.303	.130	.002	.316*	− .317*	.254	− .118	− .207	− .217
Con MEI	.364*	− .396*	.031	− .156	.219	− .302	.267	− .301	.215	.342*	.286
Con FT	− .138	.461*	− .046	.218	− .257	.479*	− .520**	.540**	− .341*	− .526**	− .409*
Con FEI	.181	.174	− .221	.115	− .196	.151	− .222	.230	− .193	− .233	− .127
MT Difference	− .005	− .073	− .187	.029	− .129	.025	− .107	.094	.072	− .160	.045
MEI Difference	.170	.044	− .126	− .054	.062	− .223	.055	− .112	.227	.240	.169
FT Difference	.035	− .186	− .041	− .075	− .078	− .014	.199	− .058	.134	− .035	.189
FEI Difference	− .070	.045	.245	.057	− .008	− .122	.158	− .101	.244	.307	.201

*US* Ultrasound, *BMI* Body Mass Index, *NYHA* New York Heart Association class, *5-STS* Five-Repetitions Sit-to Stand, *SPPB* Short Physical Performance Battery, *TUG* Timed Up and Go test, *6MWT* 6 Minute Walking Test, *UGS* Usual Gait Speed, *FGS* Fast Gait Speed, *Non-con MT* Non-contraction Muscle Thickness, *Non-con MEI* Non-contraction Muscle Echo-Intensity, *Non-con FT* Non-contraction subcutaneous Fat tissue Thickness, *Non-con FEI* Non-contraction subcutaneous Fat tissue Echo-Intensity, *Con MT* Contraction Muscle Thickness, *Con MEI* Contraction Muscle Echo-Intensity, *Con FT* Contraction subcutaneous Fat tissue Thickness, *Con FEI* Contraction subcutaneous Fat tissue Echo-Intensity, *MT Difference* Difference between contraction and non-contraction Muscle Thickness, *MEI Difference* Difference between contraction and non-contraction Muscle Echo-Intensity, *FT Difference* Difference between contraction and non-contraction subcutaneous Fat tissue Thickness, *FEI Difference* Difference between contraction and non-contraction subcutaneous Fat tissue Echo-Intensity.

*p < 0.05; **p < 0.001.

**Table 2:** Summary of models in women.

	R	R^2^	Adjusted R^2^	SE	F	*p*
5-STS	0.495	0.245	0.158	5.31	2.83	0.039
SPPB	0.569	0.324	0.247	2.03	4.20	0.007
TUG	0.561	0.315	0.237	6.90	4.03	0.009
6MWT	0.378	0.143	0.045	77.80	1.46	0.235
UGS	0.535	0.286	0.204	0.12	3.50	0.017
FGS	0.463	0.214	0.124	0.19	2.38	0.070

**Table 3:** Multivariate linear regression models in women, adjusted by age and BMI.

Dependent Outcome	Predictor Variables	Non-standardised coefficients		Typified coefficients	t	p	95%CI
		B	SE	Beta			
5-STS	(Constant)	25.746	18.689		1.378	.177	(− 12.195, 63.688)
	Non-con FT	1.486	5.277	.170	.282	.780	(− 9.227, 12.199)
	Con FT	3.258	5.307	.363	.614	.543	(− 7.516, 14.033)
	Age	− .138	.194	− .122	− .709	.483	(− .533, .257)
	BMI	− .124	.173	− .141	− .717	.478	(− .476, .227)
SPPB	(Constant)	7.655	7.145		1.071	.291	(− 6.851, 22.160)
	Non-con FT	− 3.257	2.017	− .923	− 1.614	.115	(− 7.353, .839)
	Con FT	1.143	2.029	.315	.564	.577	(− 2.976, 5.262)
	Age	.010	.074	.022	.133	.895	(− .141, .161)
	BMI	.048	.066	.134	.719	.477	(− .087, .182)
TUG	(Constant)	23.538	24.275		.970	.339	(− 25.742, 72.818)
	Non-con FT	4.677	6.854	.393	.682	.499	(− 9.237, 18.592)
	Con FT	2.911	6.893	.237	.422	.675	(− 11.083, 16.905)
	Age	− .061	.253	− .039	− .240	.812	(− .574, .452)
	BMI	− .218	.225	− .181	− .968	.340	(− .675, .239)
6MWT	(Constant)	383.865	273.635		1.403	.169	(− 171.644, 939.374)
	Non-con FT	− 57.063	77.262	− .475	− .739	.465	(− 213.914, 99.788)
	Con FT	20.286	77.704	.164	.261	.796	(− 137.462, 178.033)
	Age	− .917	2.848	− .059	− .322	.749	(− 6.698, 4.863)
	BMI	− 1.393	2.536	− .115	− .549	.586	(− 6.543, 3.756)
UGS	(Constant)	.567	.414		1.371	.179	(− .273, 1.406)
	Non-con FT	.008	.117	.041	.069	.945	(− .229, .245)
	Con FT	− .125	.117	− .609	− 1.061	.296	(− .363, .114)
	Age	− .001	.004	− .023	− .137	.892	(− .009, .008)
	BMI	.002	.004	.087	.454	.653	(− .006, .010)
FGS	(Constant)	.339	.672		.505	.617	(− 1.024, 1.703)
	Non-con FT	− .257	.190	− .837	− 1.358	.183	(− .642, .128)
	Con FT	.117	.191	.371	.616	.542	(− .270, .505)
	Age	.004	.007	.105	.595	.556	(− .010, .018)
	BMI	.003	.006	.097	.484	.632	(− .010, .016)

**Figure 1:**
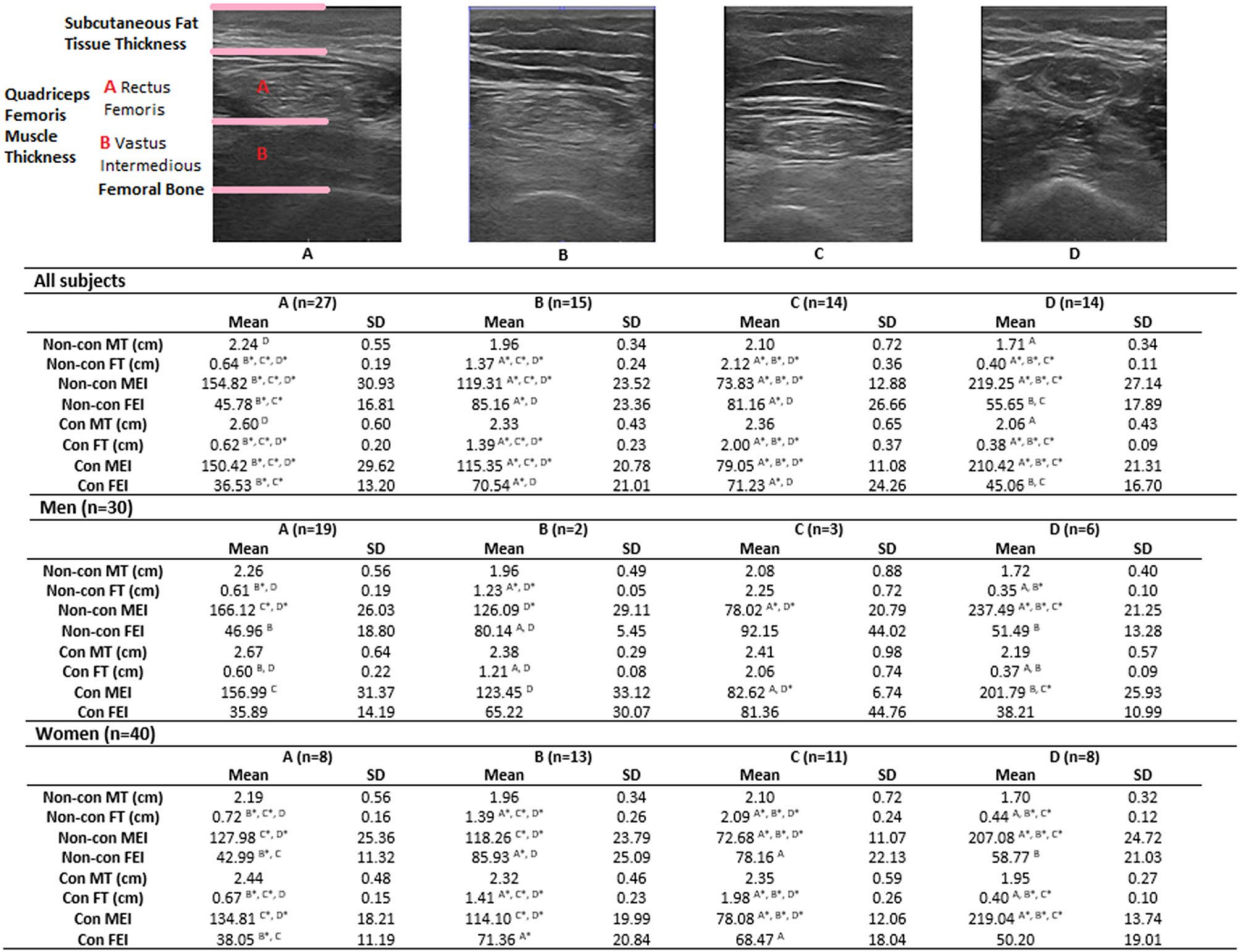
Ultrasound imagesof the four types classified based exploratory hierarchical cluster: (**A**) normal MT, FT, MEI and FEI; (**B**) increased FT, relatively decreased MT to FT, slightly high FEI and slightly low MEI; (**C**) very increased FT, normal MT, decreased MEI and slightly high FEI; (**D**) decreased FT and MT and very high MEI with normal FEI. Non-con MT: Non-concentration muscle thickness; Non-Con FT: Non-concentration subcutaneous fat tissue thickness; Non-con MEI: Non-concentration muscle echo-intensity; Non-Con FEI: Non-concentration subcutaneous fat tissue echo-intensity; Con MT: concentration muscle thickness; ConFT: concentration subcutaneous fat tissue thickness; Con-MEI: concentration muscle echo-intensity; Con-FEI: concentration subcutaneous fat tissue echo-intensity. ^A,B,C,D^*p* < 0.05; ^A*, B*, C*, D*^p < 0.001.

The factor analysis showed that the non-con and con MEI and non-con and con FT were the principal US biomarkers. Moreover, KMO values (0.548) and Bartlett’s test of sphericity (Chi-squared value = 686.295 and difference 28) (*p* < 0.001) indicated the correlation matrix was adequate for the PCA. PCA detected one factor with Eigenvalues above 1, explaining 52.75% of the variance (Supplementary Appendix G). The US biomarkers extracted by the PCA, which explained 52.75% of the variance, were used to perform an exploratory hierarchical cluster. The dendrogram was inspected to decide the number of clusters. We decided to conduct four clusters (Supplementary Appendix G). The four groups differed in non-con MEI, con MEI, non-con FT and con FT (Fig. 1). The QF characteristics of each group were (Fig. 1): (A) normal MT, FT, MEI and FEI; (B) increased FT, relatively decreased MT to FT, slightly high FEI and slightly low MEI; (C) very increased FT, normal MT, decreased MEI and slightly high FEI; (D) decreased FT and MT and very high MEI with normal FEI. Differences between the types (Supplementary Appendix H) were observed in SPPB, significantly greater in A than in C (Table 4). No physical function differences were shown between the ultrasound groups in men. In women, differences were only reported in the TUG and in the UGS between groups B and D, which were worse in group B.

**Table 4:** Comparison of each variable among the four US types classified based on exploratory hierarchical cluster.

	A (n = 27)		B (n = 15)		C (n = 14)		D (n = 14)	
	Mean	SD	Mean	SD	Mean	SD	Mean	SD
Age (years)	79.93	5.66	82.13	6.13	82.07	6.04	80.79	6.55
Height (m)	1.63	0.09	1.59	0.08	1.56	0.06	1.62	0.08
Weight (Kg)	77.24^D^	13.83	74.58	13.43	81.89^D^	17.72	67.00^A,C^	7.14
BMI (Kg/m^2^)	29.02^D^	4.77	29.48	5.01	33.60^D^	8.11	25.60^A,C^	2.10
5-STS (sec)	14.20	4.56	16.30	4.92	18.16	7.54	15.26	4.71
SPPB (0–12)	9.04^C^	2.26	7.20	2.54	6.71^A^	2.61	7.71	2.70
TUG (sec)	16.52	6.59	21.45	6.95	22.97	11.24	16.15	4.08
6MWT (m)	257.04	92.91	210.00	86.35	229.29	89.31	289.29	107.11
UGS (m/s)	0.58	0.19	0.44	0.21	0.43	0.17	0.57	0.23
FGS (m/s)	0.73	0.27	0.58	0.24	0.60	0.25	0.76	0.31
Katz Index (0–3)	0.85	0.77	0.80	0.77	1.21	1.12	1.00	1.18
Lawnton and Brody (0–4)	1.96	1.31	1.87	1.60	1.71	1.64	2.21	1.76
**NYHA (n)**								
II	17		12		10		9	
III	10		3		4		5	

	Men (n = 30)							
	A (n = 19)		B (n = 2)		C (n = 3)		D (n = 6)	
	Mean	SD	Mean	SD	Mean	SD	Mean	SD
Age (years)	79.84	5.71	78.50	12.02	88.67	7.51	76.83	7.03
Height (m)	1.68	0.06	1.72	0.08	1.64	0.02	1.69	0.04
Weight (Kg)	82.77	11.37	83.50	26.16	83.00	13.00	71.17	6.31
BMI (Kg/m^2^)	29.58	4.49	27.75	6.28	31.05	5.39	25.01	2.04
5-STS (sec)	14.14	4.67	11.59	2.20	11.63	0.56	17.45	5.88
SPPB (0–12)	9.37	2.41	11.00	0	9.33	2.08	6.50	2.95
TUG (sec)	15.53	7.01	11.50	0.80	12.62	2.64	16.51	5.77
6MWT (m)	269.74	102.88	307.50	10.61	310.00	56.79	312.50	145.87
UGS (m/s)	0.62	0.21	0.87	0.13	0.63	0.24	0.61	0.35
FGS (m/s)	0.78	0.28	0.92	0.12	0.86	0.37	0.78	0.46
Katz Index (0–3)	0.79	0.79	0	0	0.67	0.58	1.00	1.26
Lawnton and Brody (0–4)	1.84	1.30	3.00	1.41	1.67	2.08	1.50	1.64
**NYHA (n)**								
II	13		1		2		3	
III	6		1		1		3	

	Women (n = 40)							
	A (n = 8)		B (n = 13)		C (n = 11)		D (n = 8)	
	Mean	SD	Mean	SD	Mean	SD	Mean	SD
Age (years)	80.13	5.91	82.69	5.41	80.27	4.43	83.75	4.59
Height (m)	1.53	0.04	1.57	0.06	1.55	0.05	1.57	0.06
Weight (Kg)	64.12	9.91	73.31	11.75	81.59	19.34	63.87	6.33
BMI (Kg/m^2^)	27.69	5.46	29.75	5.05	34.29 ^D^	8.79	26.03 ^C^	2.17
5-STS (sec)	14.32	4.58	17.02	4.86	19.94	7.59	13.62	3.06
SPPB (0–12)	8.25	1.75	6.62	2.18	6.00	2.32	8.63	2.26
TUG (sec)	18.87	5.07	22.98^D^	6.11	25.80	11.05	15.89 ^B^	2.65
6MWT (m)	226.88	58.06	195.00	82.84	207.27	85.07	271.88	72.80
UGS (m/s)	0.47	0.11	0.38^D^	0.13	0.37^D^	0.11	0.54^B,C^	0.12
FGS (m/s)	0.61	0.21	0.53	0.21	0.53	0.17	0.74	0.18
Katz index (0–3)	1.00	0.76	0.92	0.76	1.36	1.21	1.00	1.19
Lawnton and Brody (0–4)	2.25	1.39	1.69	1.60	1.73	1.62	2.75	1.75
**NYHA (n)**								
II	4		11		8		6	
III	4		2		3		2	

(A) normal MT, FT, MEI and FEI; (B) increased FT, relatively decreased MT to FT, slightly high FEI and slightly low MEI; (C) very increased FT, normal MT, decreased MEI and slightly high FEI; (D) decreased FT and MT and very high MEI with normal FEI

*US* Ultrasound, *BMI* Body Mass Index, *5-STS* Five-Repetitions Sit-to Stand, *SPPB* Short Physical Performance Battery, *TUG* Timed Up and Go test, *6MWT* 6 Minute Walking Test, *UGS* Usual Gait Speed, *FGS* Fast Gait Speed, *NYHA* New York Heart Association class, *SD* Standard Deviation.

^A,B,C,D^*p* < 0.05; ^A*, B*, C*, D*^p < 0.001.

## Discussion

Our results reported that the non-con FT, non-con FEI, con FT and con FEI were significantly greater in women than in men. Ageing has been associated with a greater increase of subcutaneous FT in women than in men^24^. Our results also showed that women with HFpEF have worse physical functional performance in SPPB, TUG and 6MWT and slower UGS and FGS than men. One of the causes of this impaired physical function in women may be the subcutaneous FT because non-con FT and con FT correlated in women with muscle strength and physical function. This correlation ranged from poor between the non-con FT and the 6MWT (r = − 0.365, *p* = 0.021) to moderate between the non-con FT and the SPPB (r = − 0.555, *p* < 0.001). Abdominal FT, thigh FT and intermuscular fat have been related to worse VO2 peak or exercise intolerance in patients with HFpEF^25^. Ageing, increased oxidative stress, and low-grade chronic inflammation are three related and age-dependent processes that contribute to the development of age-related chronic diseases and the loss of physical function, muscle strength and muscle mass^26^. Sarcopenia induces metabolic and endocrine abnormalities that could increase the risk of developing an HFpEF^27^. Low-grade chronic inflammation is strongly associated with increased body fat mass because adipose tissue produces pro-inflammatory cytokines^28^. In this way, FT could accelerate the physical function decline in patients with HFpEF. However, there is controversy about the prognostic role of fat tissue in patients with HF or older adults. While FT has been associated with a greater risk of HF among older adults, especially in those with diabetes^29^, greater fat and muscle mass were associated with lower mortality risk in patients with HF^30^.

Previous literature has found accentuated muscle dysfunction, reduced mitochondrial size in skeletal muscle, increased levels of atrophy genes and proteins and metabolic abnormalities in skeletal muscle in stable outpatients with HFpEF compared with older adults with HFrEF and healthy controls^31^. Like fat mass, greater muscle mass has been related to a better prognosis in patients with HF^30^. Contrary to fat mass, higher muscle mass was related to better VO2 peak and better physical function in patients with HF^25^. Older adults with HFpEF showed a non-con QF MT of 2.05 cm. Nakano et al.^32^ showed that patients with HF have a reduced QF MT compared with healthy people. Thus, older adults with HFpEF seem to have a reduced MT, which could be due to factors such as age or gender because muscle mass loss has been related to ageing^33^ and is more quickly in women than in men^34^. Our results did not show an interesting relationship between MT and physical function in men or women with HFpEF. Some studies showed a relationship between QF MT and QF MEI assessed by US and muscle strength and physical function in older adults^13^. However, Ticinesi et al.^35^ pointed out that the relationship between MT and MEI assessed by US and muscle strength or physical function differs among studies. These differences among studies could be avoided by standardising the US measurements^12,36^.

Older adults with HFpEF also showed reduced muscle strength, decreased physical functional performance, and slowed gait speed. A previous study also reported that older adults with HFpEF have limited functional aerobic capacity and poor physical function^37^. Worse muscle strength and physical functional performance have been reported with ageing and in women^33,38^. Physical functional performance was related to prognosis in patients with HF^39^. Moreover, older adults with HFpEF used to be difficult to manage due to physical function impairment and a lack of response to most medical treatments^40^. In the present study, non-con FT and con FT explained 24.5% of the 5-STS variance, 32.4% of the SPPB variance, 31.5% of the TUG variance, 21.4% of the FGS variance, and 28.6% of the UGS variance in women. However, these results seem reasonable because the physical function is a multidimensional construct. Thus, fat mass, intermuscular and intramuscular fat infiltration, muscle mass, muscle contractile properties, muscle strength, and nervous system functionality have been related to physical function and disability^41,42^. However, these variables can only explain up to 40% of the physical function variance on their own^41–43^. Thus, the US assessment of the musculoskeletal structural biomarkers should not replace the physical function assessment. However, the US and physical function assessments could help clinicians properly manage a complex and heterogeneous group of older adults with HFpEF. Therapeutic exercise improves physical function and muscle strength in older adults^44^. Therapeutic exercise can improve muscle mass, reduce FT, body fat mass and intramuscular and intermuscular fat infiltration, above all when exercise is performed at high intensity^45,46^. Some drugs taken regularly may interact with mechanisms that can alter the balance between protein synthesis and degradation^47^. In this way, these drugs may show a harmful or a beneficial effect on muscle mass, muscle strength and physical function^47^. The US has all the advantages to be used in clinical practice to assess musculoskeletal structural biomarkers and monitor the effect of clinical interventions^12^. We conducted a classification of older adults with HFpEF based on their US biomarkers. Physical functional differences in the SPPB were observed between groups A and C, while differences in the TUG and UGS were reported between groups B and D in women. Kawai et al.^13^ also conducted a classification based on US biomarkers of the QF muscle that could assess muscle strength, physical function, and sarcopenia in community-dwelling older adults. The four groups that Kawai et al.^13^ showed similarities with the groups formed in this study. Thus, the groups in this study could be classified as (A): "normal type"; (B) "sarcopenic obesity type"; (C) "obesity type"; (D) "sarcopenia type". However, older adults with HFpEF show lower non-con MT, higher non-con FT, lower non-con FEI and higher non-con MEI than older adults included in Kawai et al.^13^ study. Thus, older adults with HFpEF seem to have a lower muscle contractile component and a more significant increase in intramuscular and intermuscular fat tissue than healthy older adults. However, the differences could be due to the small sample size of our study. Kawai et al.^13^ did not show the QF characteristics in a con situation. US assessment of con MT has shown to be an objective measure superior to the assessment of non-con MT^48^.

### Implications for clinical practice

Our results showed a relationship between subcutaneous FT and muscle strength and physical function in older adults with HFpEF, especially in women with HFpEF. The US allows assessing musculoskeletal structural biomarkers with high resolution within a relatively short period and has all the potential to be used in clinical practice to assess muscle architecture^11,12^. Clinicians should incorporate US assessment into their clinical practice since US assessment could allow them to monitor the effectiveness of clinical interventions. Thus, US assessment allows clinicians to monitor if they are applying therapeutic exercise at the intensities necessary to obtain structural changes in the musculoskeletal system or if the prescribed drugs have a beneficial or harmful effect on musculoskeletal structural biomarkers.

### Future research

Future studies should analyse US biomarkers differences between older adults with HFpEF and older adults with HFrEF or healthy people. Future studies could assess US biomarkers differences between both legs. Future studies should determine the responsiveness of the US assessment. Future studies should confirm the findings shown by the present study, including a larger sample size.

### Strengths and limitations of the study

Our study was the first study assessing the relationship between US biomarkers of the QF muscle and subcutaneous fat tissue with gender, age, BMI, muscle strength and physical function in older adults with HFpEF. The author IJF-A also conducted all the measurements to reduce the risk of bias among sonographers. Moreover, the US is a tool with good intra-rater reliability^15^ and excellent inter-rater reliability, regardless of the sonographer's experience level, the severity of the patient illness, or the patient setting^49^. All the US measurements were performed with the same US at the same point of the quadriceps femoris and with the same US parameters. The older adults were also placed in the same chair and posture to avoid biases when obtaining the US biomarkers. The statistically significant and non-significant results were presented to avoid publication bias. However, several limitations must be taken into account when interpreting the results. The US image landmark and the older adults’ position may have affected the US biomarkers and the correlations shown in our study^36^. The included patients’ age (mean = 81.00 years old) may also have affected the magnitude of the correlation since older age was associated with a lower correlation^50^. We have only performed the US assessment on the right leg, but there could be differences between both legs. The morphological and clinical characteristics of the included older adults with HFpEF could have affected the US biomarkers, causing a detection bias in these outcomes. The sample size is insufficient to perform a US stratification.

## Conclusions

Non-con FT and con FT correlate with women's muscle strength and physical function, so structural musculoskeletal biomarkers assessed by the US could be relevant biomarkers related to muscle strength and physical function in older adults with HFpEF. These US biomarkers could also facilitate the management of older adults with HFpEF and assess the effectiveness of treatments on the musculoskeletal structure.

## Supplementary Information

Supplementary Information.

## Data Availability

The data that support the findings of this study are available from the corresponding author, [AICV], upon reasonable request.

## Abbreviations

HF: Heart failure
HFpEF: Heart failure with preserved ejection fraction
HFrEF: Heart failure with reduced ejection fraction
SPPB: Short physical performance battery
TUG: Timed up and go test
SMM: Skeletal muscle mass
ASM: Appendicular skeletal muscle mass
MRI: Magnetic resonance imaging
CT: Computed tomography
US: Ultrasound
QF: Quadriceps femoris muscle
BMI: Body mass index
NYHA: New York Heart Association class
MMSE: Mini-mental state examination
STROBE: Strengthening the reporting of observational studies in epidemiology statement
MT: Muscle thickness
ICC: Intraclass correlation coefficient
FT: Subcutaneous fat tissue thickness
Non-con: Non-contraction muscle situation
Con: Contraction muscle situation
MEI: Muscle echo-intensity
FEI: Subcutaneous fat tissue echo-intensity
5-STS: Five-repetitions sit-to-stand
6MWT: Six-minute walking test
UGS: Usual pace gait speed
FGS: Fast pace gait speed
aCGA: Abbreviated comprehensive geriatric
SD: Standard deviation
t-test: Student's t-test
r: Pearson correlation coefficient
ρ: Spearman's rho
R^2^: Coefficient of determination
PCA: Principal component analysis
KMO: Kaiser–Meyer–Oklin
ANOVA: One-way analysis of variance
SPSS: Statistical Package for the Social Sciences
