# Supplementary material for: Relationship between quadriceps femoris muscle architecture and muscle strength and physical function in older adults with heart failure with preserved ejection fraction
Source: Sci Rep. 2022 Dec 15;12:21660. doi: 10.1038/s41598-022-26064-7 (PMC9755120; doi:10.1038/s41598-022-26064-7)
Supplement: Supplementary file 1 — Supplementary Information. [file 41598_2022_26064_MOESM1_ESM.docx]

**Supplementary Appendix A**. STROBE Statement—Checklist of items that should be included in reports of *cross-sectional studies*

|  | Item No | Recommendation | Page No |
| --- | --- | --- | --- |
| **Title and abstract** | 1 | (*a*) Indicate the study’s design with a commonly used term in the title or the abstract | 1 |
|  |  | (*b*) Provide in the abstract an informative and balanced summary of what was done and what was found | 2 |
| Introduction | | | |
| Background/rationale | 2 | Explain the scientific background and rationale for the investigation being reported | 4-5 |
| Objectives | 3 | State specific objectives, including any prespecified hypotheses | 5 |
| Methods | | | |
| Study design | 4 | Present key elements of study design early in the paper | 5 |
| Setting | 5 | Describe the setting, locations, and relevant dates, including periods of recruitment, exposure, follow-up, and data collection | 5 |
| Participants | 6 | (*a*) Give the eligibility criteria, and the sources and methods of selection of participants | 5 |
| Variables | 7 | Clearly define all outcomes, exposures, predictors, potential confounders, and effect modifiers. Give diagnostic criteria, if applicable | 6-9 |
| Data sources/ measurement | 8* | For each variable of interest, give sources of data and details of methods of assessment (measurement). Describe comparability of assessment methods if there is more than one group | 6-9 |
| Bias | 9 | Describe any efforts to address potential sources of bias | 21-22 |
| Study size | 10 | Explain how the study size was arrived at | 9 |
| Quantitative variables | 11 | Explain how quantitative variables were handled in the analyses. If applicable, describe which groupings were chosen and why | 10-11 |
| Statistical methods | 12 | (*a*) Describe all statistical methods, including those used to control for confounding | 10-11 |
|  |  | (*b*) Describe any methods used to examine subgroups and interactions | N/A |
|  |  | (*c*) Explain how missing data were addressed | N/A |
|  |  | (*d*) If applicable, describe analytical methods taking account of sampling strategy | 10-11 |
|  |  | (*e*) Describe any sensitivity analyses | N/A |
| Results | | | |
| Participants | 13* | (a) Report numbers of individuals at each stage of study—eg numbers potentially eligible, examined for eligibility, confirmed eligible, included in the study, completing follow-up, and analysed | 11 |
|  |  | (b) Give reasons for non-participation at each stage | N/A |
|  |  | (c) Consider use of a flow diagram | N/A |
| Descriptive data | 14* | (a) Give characteristics of study participants (eg demographic, clinical, social) and information on exposures and potential confounders | 11  Appendix D and E |
|  |  | (b) Indicate number of participants with missing data for each variable of interest | 11 |
| Outcome data | 15* | Report numbers of outcome events or summary measures | 11, 13, 15  Appendix D, E, F, Table 1, 2, 3 4 and Figure 1 |
| Main results | 16 | (*a*) Give unadjusted estimates and, if applicable, confounder-adjusted estimates and their precision (eg, 95% confidence interval). Make clear which confounders were adjusted for and why they were included | 11, 13, 15  Appendix D, E, F, G, H, Table 1, 2, 3 4 and Figure 1 |
|  |  | (*b*) Report category boundaries when continuous variables were categorized | N/A |
|  |  | (*c*) If relevant, consider translating estimates of relative risk into absolute risk for a meaningful time period | N/A |
| Other analyses | 17 | Report other analyses done—eg analyses of subgroups and interactions, and sensitivity analyses | N/A |
| Discussion | | | |
| Key results | 18 | Summarise key results with reference to study objectives | 18-20 |
| Limitations | 19 | Discuss limitations of the study, taking into account sources of potential bias or imprecision. Discuss both direction and magnitude of any potential bias | 21-22 |
| Interpretation | 20 | Give a cautious overall interpretation of results considering objectives, limitations, multiplicity of analyses, results from similar studies, and other relevant evidence | 18-21 |
| Generalisability | 21 | Discuss the generalisability (external validity) of the study results | N/A |
| Other information | | | |
| Funding | 22 | Give the source of funding and the role of the funders for the present study and, if applicable, for the original study on which the present article is based | 22 |

*Give information separately for exposed and unexposed groups.

**Note:** An Explanation and Elaboration article discusses each checklist item and gives methodological background and published examples of transparent reporting. The STROBE checklist is best used in conjunction with this article (freely available on the Web sites of PLoS Medicine at http://www.plosmedicine.org/, Annals of Internal Medicine at http://www.annals.org/, and Epidemiology at http://www.epidem.com/). Information on the STROBE Initiative is available at www.strobe-statement.org.


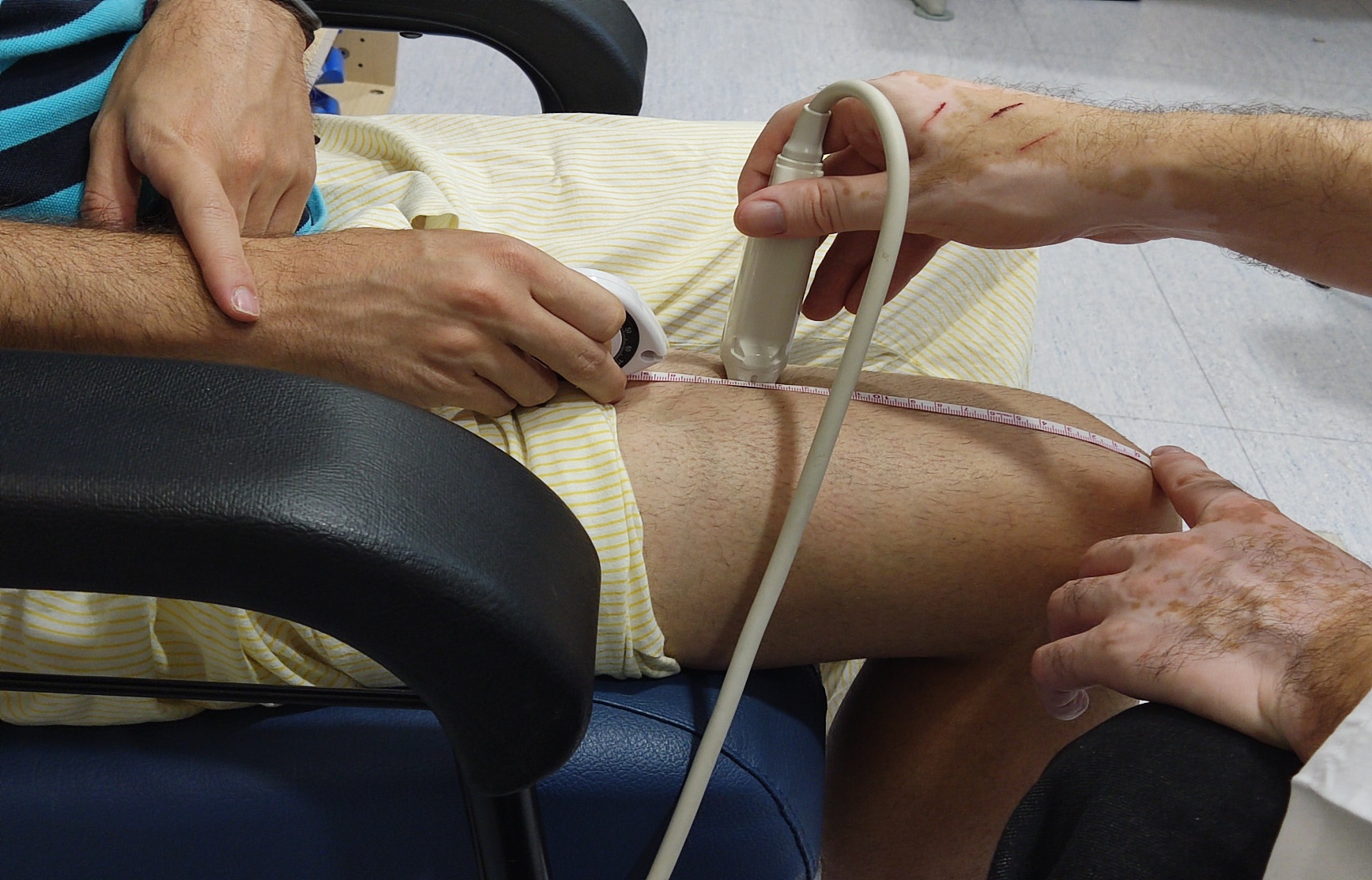


**Supplementary Appendix B.** The landmark and the patients´ position for the ultrasound assessment.


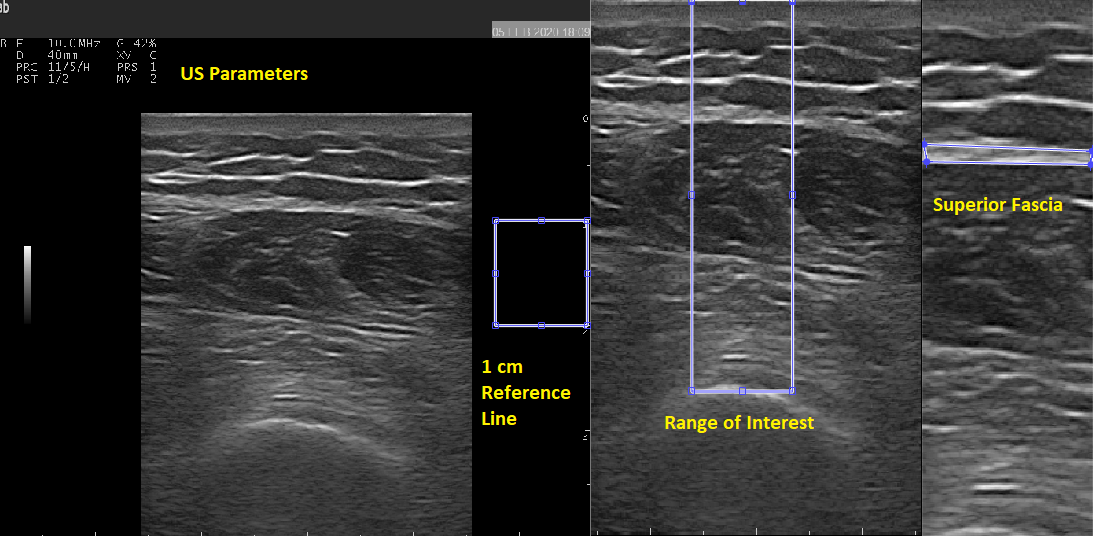


**Supplementary Appendix C**. Range of interest and selected areas.

| **Supplementary Appendix D.** Descriptive statistics of the study outcomes and outcomes differences between gender (n=70). | | | | | |
| --- | --- | --- | --- | --- | --- |
|  | **All Older Adults (n=70)** | **Men (n=30, 42.90%)** | **Women (n=40, 57.10%)** |  |  |
|  | **Mean (SD)** | **Mean (SD)** | **Mean (SD)** | **Mean Difference Between Gender (SE)** | **P Between Gender** |
| **Clinical** |  |  |  |  |  |
| Age (years) | 81.00 (5.97) | 80.03 (6.90) | 81.73 (5.13) | -1.69 (1.50) | 0.264 |
| **Antropometric** |  |  |  |  |  |
| Height (m) | 1.61 (0.08) | 1.68 (0.06) | 1.55 (0.05) | 0.12 (0.01) | <0.001** |
| Weight (Kg) | 75.55 (14.20) | 80.52 (12.04) | 71.83 (14.69) | 8.69 (3.29) | 0.010* |
| BMI (Kg/m^2^) | 29.35 (5.78) | 28.69 (4.54) | 29.84 (6.58) | -1.15 (1.40) | 0.415 |
| **US biomarkers** |  |  |  |  |  |
| **QF Muscle** |  |  |  |  |  |
| Non-con MT (cm) | 2.05 (0.55) | 2.12 (0.58) | 1.99 (0.52) | 0.12 (0.13) | 0.353 |
| Non-con MEI | 133.59 (49.74) | 161.28 (46.55) | 111.59 (40.81) | 49.68 (11.20) | <0.001** |
| Con MT (cm) | 2.39 (0.57) | 2.53 (0.65) | 2.28 (0.49) | 0.25 (0.14) | 0.067 |
| Con MEI | 130.50 (44.32) | 151.59 (41.98) | 113.75 (39.12) | 37.84 (10.41) | 0.001* |
| MT Difference (cm) | 0.34 (0.29) | 0.42 (0.31) | 0.29 (0.26) | 0.13 (0.07) | 0.064 |
| MEI Difference | -3.09 (30.00) | -9.69 (35.77) | 2.16 (23.75) | -11.84 (8.00) | 0.146 |
| **Subcutaneous Fat Tisuue** |  |  |  |  |  |
| Non-con FT (cm) | 1.05 (0.68) | 0.76 (0.60) | 1.26 (0.66) | -0.50 (0.15) | 0.002* |
| Non-con FEI | 63.27 (26.68) | 54.60 (24.81) | 69.77 (26.47) | -15.18 (6.23) | 0.017* |
| Con FT (cm) | 1.01 (0.65) | 0.74 (0.56) | 1.22 (0.64) | -0.47 (0.14) | 0.002* |
| Con FEI | 52.46 (23.91) | 42.85 (23.22) | 59.67 (22.05) | -16.82 (5.45) | 0.003* |
| FT Difference (cm) | -0.03 (0.14) | -0.02 (0.10) | -0.04 (0.17) | 0.02 (0.03) | 0.489 |
| FEI Difference | -10.81 (13.85) | -11.74 (14.11) | -10.10 (13.79) | -1.64 (3.36) | 0.628 |
| **Muscle Strength** |  |  |  |  |  |
| 5-STS (sec) | 15.65 (5.46) | 14.38 (4.80) | 16.60 (5.79) | -2.22 (1.30) | 0.093 |
| **Physical Functional Performance** |  |  |  |  |  |
| SPPB (0-12) | 7.91 (2.61) | 8.90 (2.66) | 7.18 (2.34) | 1.72 (0.60) | 0.005* |
| TUG (sec) | 18.79 (7.85) | 15.17 (6.23) | 21.51 (7.90) | -6.35 (1.75) | 0.001* |
| 6MWT (m) | 247.86 (95.88) | 284.83 (104.27) | 220.13 (79.62) | 64.71 (21.97) | 0.004* |
| UGS (m/s) | 0.52 (0.21) | 0.63 (0.24) | 0.43 (0.13) | 0.21 (0.05) | <0.001** |
| FGS (m/s) | 0.68 (0.27) | 0.80 (0.31) | 0.59 (0.20) | 0.21 (0.06) | 0.002* |
| **Self-reported questionnaires** |  |  |  |  |  |
| Katz Index (0-3) | 0.94 (0.93) | 0.77 (0.86) | 1.08 (0.97) | -0.31 (0.22) | 0.172 |
| Lawton & Brody (0-4) | 1.94 (1.51) | 1.83 (1.42) | 2.03 (1.59) | -0.19 (0.37) | 0.603 |
|  | **n (%)** |  |  |  |  |
| **Clinical**  **NYHA**  II  III | 48 (68.60%)  22 (31.40%) | 19 (63.30%)  11 (36.70%) | 29 (72.50%)  11 (27.50%) |  | 0.414  0.414 |
| **SD**: Standard Deviation; **SE**: Standard Error; **BMI**: Body Mass Index; **US**: Ultrasound; **QF**: Quadriceps Femoris; **Non-con MT**: Non-contraction Muscle Thickness; **Non-con MEI**: Non-contraction Muscle Echo-Intensity; **Con MT**: Contraction Muscle Thickness; **Con MEI**: Contraction Muscle Echo-Intensity; **MT Difference**: Difference between contraction and non-contraction Muscle Thickness; **MEI Difference**: Difference between contraction and non-contraction Muscle Echo-Intensity; **Non-con FT**: Non-contraction subcutaneous Fat tissue Thickness; **Non-con FEI**: Non-contraction subcutaneous Fat tissue Echo-Intensity; **Con FT**: Contraction subcutaneous Fat tissue Thickness; **Con FEI**: Contraction subcutaneous Fat tissue Echo-Intensity; **FT Difference**: Difference between contraction and non-contraction subcutaneous Fat tissue Thickness; **FEI Difference**: Difference between contraction and non-contraction subcutaneous Fat tissue Echo-Intensity; **5-STS**: Five-Repetitions Sit-to Stand; **SPPB**: Short Physical Performance Battery; **TUG**: Timed Up and Go test; **6MWT**: 6 Minute Walking Test; **UGS**: Usual Gait Speed; **FGS**: Fast Gait Speed; **NYHA**: New York Heart Association class.  ***p<0.05; **p<0.001** | | | | | |

**Supplementary Appendix E.** Participants' clinical-epidemiological variables and blood and urinary biomarkers (n=70).

|  | | **Mean (SD)** | | | | **Min-Max** |
| --- | --- | --- | --- | --- | --- | --- |
| LVEF (%) | | 60.50 (4.44) | | | | 50.0-75.0 |
| Comorbidities (n) | | 8.31 (1.92) | | | | 4.0-14.0 |
| Drugs/Day (n) | | 10.16 (3.19) | | | | 3.0-19.0 |
| LAD (mm) | | 42.23 (5.60) | | | | 28-56 |
| LVESD (mm) | | 29.48 (5.05) | | | | 22.0 45.0 |
| LVEDD (mm) | | 47.79 (3.78) | | | | 40.0-59.0 |
| IST (mm) | | 12.14 (1.83) | | | | 7.0-16.0 |
| IST, Posterior Wall (mm) | | 11.16 (1.54) | | | | 7.0-15.0 |
| **Blood Biomarkers** | |  | | | | |
| Hb (g/dL) | | 13.99 (11.55) | | 6.40-108.0 | | |
| MCV (fL) | | 95.70 (19.76) | | 72-247 | | |
| Leukocytes Count (x10^9^/L) | | 8.15 (4.96) | | 3.54-45.20 | | |
| Blood Platelets (x10^9^/L) | | 219.54 (68.55) | | 8-415 | | |
| Glucose (mg/dL) | | 102.47 (28.80) | | 61-195 | | |
| Creatinine (mg/dL) | | 1.37 (0.84) | | 0.51-6.33 | | |
| GF (mL/min/1,73 m^2^) | | 50.63 (20.59) | | 8-90 | | |
| Na^+^ (mEq/L) | | 139.67 (3.35) | | 124-147 | | |
| K^+^ (mEq/L) | | 4.64 (0.46) | | 3.5-5.8 | | |
| Cl^-^ (mEq/L) | | 101.39 (4.22) | | 89-111 | | |
| Glycosylated hemoglobin (%) | | 6.41 (1.15) | | 4.8-10.4 | | |
| Ferritin (ng/mL) | | 127.56 (144.01) | | 10-738 | | |
| Transferrin Saturation (%) | | 21.59 (26.34) | | 5-214 | | |
| Total Cholesterol (mg/dL) | | 162.13 (42.19) | | 98-317 | | |
| LDL (mg/dL) | | 87.40 (29.73) | | 41-167 | | |
| HDL (mg/dL) | | 46.44 (13.78) | | 28-90 | | |
| NT-proBNP (pg/mL) | | 2331.25 (3621.81) | | 42-19118 | | |
| Vitamin D (ng/mL) | | 23.82 (10.68) | | 7-50 | | |
| Vitamin B12 (pg/mL) | | 506.71 (670.49) | | 103-4668 | | |
| ALT (U/L) | | 21.82 (13.62) | | 8-94 | | |
| CA-125 Antigen (U/mL) | | 41.09 (86.36) | | 2-525 | | |
| Folic Acid (ng/mL) | | 6.67 (3.76) | | 3-13 | | |
| Thyrotropin (µIU/mL) | | 2.35 (1.74) | | 0.1-12 | | |
| Albumin (g/dL) | | 4.03 (4.02) | | 2.30-36 | | |
| Bilirubin (mg/dL) | | 0.61 (0.31) | | 0.2-1.8 | | |
| **Urinary Biomarkers** | | |  |  | | |
| Urine Creatinine (mg/dL) | | | 79.31 (32.35) | 36.00-141.00 | | |
|  | **n (Percentage)** | | | |  | |
| **Drugs** |  | | | |  | |
| ACE Inhibitors  ARB  Beta-Blockers  Ivabradine  Ca-antagonists  Loop Diuretics  Mineralocorticoid Receptor Antagonist  Thiazide  Nitrates  Acarboxyprothrombin  Factor Xa Inhibitors  Acetylsalicylic Acid  Hypoglycemic Agents  Metformin  SGT2I  GLP1  DPP4  Insulin | | 14 (20.00%)  44 (62.90%)  53 (75.70%)  4 (5.70%)  20 (28.60%)  60 (85.71%)  21 (30.00%)  9 (12.90%)  17 (24.30%)  11 (15.70%)  29 (41.40%)  25 (35.70%)  33 (47.10%)  16 (22.90%)  8 (11.40%)  2 (2.90%)  18 (25.70%)  20 (28.60%) | | | |  |
| **Comorbidities** | |  | | | |  |
| Arterial Hypertension | | 68 (97.10%) | | | |  |
| DM | | 40 (57.10%) | | | |  |
| Dyslipidemias | | 60 (85.70%) | | | |  |
| [Atrial Fibrillation](https://www.ncbi.nlm.nih.gov/mesh/68001281) | | 41 (58.60%) | | | |  |
| COPD | | 20 (28.60%) | | | |  |
| Stroke | | 9 (12.90%) | | | |  |
| CRI | | 45 (64.30%) | | | |  |
| OSA | | 15 (21.40%) | | | |  |
| Cognitive Impairment | | 8 (11.40%) | | | |  |
| Anemia | | 32 (45.70%) | | | |  |
| PAD | | 4 (5.70%) | | | |  |
| Depression | | 21 (30.00%) | | | |  |
| Cancer Disease | | 10 (14.30%) | | | |  |
| Heart Valve Disease | | 46 (65.70%) | | | |  |
| Aortic Valve Disease | | 25 (35.70%) | | | |  |
| Mitral Valve Disease | | 32 (45.70%) | | | |  |
| Tricuspid Valve Disease | | 17 (24.30%) | | | |  |
| Pulmonary Hypertension | | 12 (17.10%) | | | |  |
| **BMI**  Normal Weight  Overweight  Obesity | | 16 (22.90%)  29 (41.40%)  25 (35.70%) | | | |  |
| **Gender**  female  male | | 40 (57.10%)  30 (42.90%) | | | |  |
| **NYHA**  II  III | | 48 (68.60%)  22 (31.40%) | | | |  |
| **History of Smoking**  No  Yes | | 49 (61.47%)  27 (38.57%) | | | |  |
| **History of Alcohol**  No  Yes | | 64 (91.43%)  6 (8.57%) | | | |  |
| **Number of falls in the last year**  0  1  2  3  > 3 | | 30 (42.90%)  20 (28.60%)  8 (11.40%)  6 (8.60%)  6 (8.60%) | | | |  |
| **Marital Status**  Single  Married  Divorced  Widower | | 6 (8.60%)  27 (38.60%)  1 (1.40%)  36 (51.40%) | | | |  |
| **Academic Degree/Studies Level**  Not knowing how to read or write  Literacy  Primary studies  Secondary studies  Higher education | | 10 (14.30%)  32 (45.70%)  12 (17.10%)  6 (8.60%)  10 (14.30%) | | | |  |

**LVEF:** Left Ventricular Ejection Fraction; **LAD:** Left Atrial Dimension; **LVESD:** Left Ventricular End-Systolic Dimension; **LVEDD:** Left Ventricular End-Diastolic Dimension; **IST:** Interventricular Septum Thickness; **Hb**: Hemoglobin; **MCV**: Mean Corpuscular Volume; **GF**: Glomerular Filtering; **Na^+^**: Sodium; **K^+^**: Potassium; **Cl^-^**: Chlorine; **LDL**: Low-Density Lipoproteins Cholesterol; **HDL**: High-Density Lipoproteins Cholesterol; **NT-proBNP**: pro-Brain Natriuretic Peptide; **ALT**: Alanine Aminotransferase; **CA-125 Antigen**: Carbohydrate Antigen; **ACE inhibitors**: Angiotensin-Converting Enzyme Inhibitors; **ARB**: Angiotensin II Receptor Blocker; **SGT2I**: Sodium-Glucose Transporter 2 Inhibitors; **GLP1**: Glucagon-Like Peptide 1; **DPP4**: DiPeptidyl Peptidase-4; **DM**: Diabetes Mellitus; **COPD**: Chronic Obstructive Pulmonary Disease; **CRI**: Chronic Renal Insufficiency; **OSA**: Obstructive Sleep Apnea; **PAD**: Peripheral Arterial Disease; **LVH**: Left Ventricular Hypertrophy; **LVD**: Left Ventricular Dilatation; **LAD**: Left Atrial Dilatation; **BMI**: Body Mass Index; **NYHA**: New York Heart Association class; **SD**: Standard Deviation.

| **Supplementary Appendix F.** Partial correlation coefficients after controlling for BMI between the US biomarkers and self-reported questionnaires, muscle strength and physical functional performance, stratified by gender. | | | | | | | | | |
| --- | --- | --- | --- | --- | --- | --- | --- | --- | --- |
|  | **NYHA** | **Katz Index** | **Lawnton & Brody** | **5-STS** | **SPPB** | **TUG** | **6MWT** | **UGS** | **FGS** |
| **Men (n=30)** | | | | | | | | | |
| **Non-con MT** | .006 | -.047 | **.426*** | .111 | -.017 | .304 | **-.417*** | -.249 | -.092 |
| **Non-con MEI** | .021 | -.010 | .185 | .244 | -.359 | .183 | -.105 | -.266 | .015 |
| **Non-con FT** | .022 | -.067 | .060 | -.363 | .309 | -.313 | .260 | .223 | .296 |
| **Non-con FEI** | .153 | -.119 | -.092 | -.260 | .164 | -.347 | **427*** | .240 | .222 |
| **Con MT** | -.137 | .094 | **.416*** | -.021 | .095 | .209 | -.189 | -.098 | .141 |
| **Con MEI** | .104 | .073 | -.271 | .283 | -.295 | .194 | -.218 | -.286 | .014 |
| **Con FT** | .081 | -.134 | .093 | -.324 | .278 | -.290 | .275 | .245 | .265 |
| **Con FEI** | .248 | -.245 | -.193 | -.080 | .013 | -.194 | .216 | .106 | .036 |
| **MT Difference** | -.265 | .256 | .041 | -.230 | .205 | -.134 | **.366*** | .246 | **.419*** |
| **MEI Difference** | .082 | .090 | **-.524*** | -.015 | .151 | -.031 | -.094 | .041 | -.004 |
| **FT Difference** | .270 | -.293 | .129 | .356 | -.289 | .254 | -.048 | .004 | -.284 |
| **FEI Difference** | .140 | -.195 | -.157 | .325 | -.268 | .292 | **-.395*** | -.248 | -.332 |
| **Women (n=40)** | | | | | | | | | |
| **Non-con MT** | **.426*** | .063 | .032 | .253 | -.208 | .163 | -,096 | -.079 | -.189 |
| **Non-con MEI** | .025 | -.032 | .130 | -.114 | .183 | -.212 | -.015 | .179 | .129 |
| **Non-con FT** | -.056 | .145 | -.263 | **.448*** | **-.541**** | **.544**** | -.290 | **-.493**** | **-.407*** |
| **Non-con FEI** | **-.345*** | .046 | -.235 | .173 | -.251 | .231 | -.268 | **-.341*** | -.192 |
| **Con MT** | **.340*** | .104 | -.057 | .291 | -.289 | .232 | -.071 | -.180 | -.183 |
| **Con MEI** | .003 | -.081 | .252 | -.272 | .230 | -.297 | .145 | **.347*** | .251 |
| **Con FT** | -.052 | .149 | -.288 | **.458*** | **-.497**** | **.540**** | -.265 | **-.516**** | **-.365*** |
| **Con FEI** | -.236 | .116 | -.242 | .125 | -.196 | .210 | -.157 | -.210 | -.096 |
| **MT Difference** | -.205 | .066 | -.163 | .038 | -.124 | .106 | .056 | -.174 | .032 |
| **MEI Difference** | -.034 | -.072 | .176 | -.231 | .062 | -.116 | .242 | .244 | .177 |
| **FT Difference** | .018 | .009 | -.079 | .019 | .170 | -.032 | .093 | -.067 | .158 |
| **FEI Difference** | .286 | .095 | .067 | -.132 | .170 | -.108 | .263 | **.319*** | .215 |
| **BMI**: Body Mass Index; **US**: Ultrasound; **NYHA**: New York Heart Association class; **5-STS**: Five-Repetitions Sit-to Stand; **SPPB**: Short Physical Performance Battery; **TUG**: Timed Up and Go test; **6MWT**: 6 Minute Walking Test; **UGS**: Usual Gait Speed; **FGS**: Fast Gait Speed; **Non-con MT**: Non-contraction Muscle Thickness; **Non-con MEI**: Non-contraction Muscle Echo-Intensity; **Non-con FT**: Non-contraction subcutaneous Fat tissue Thickness; **Non-con FEI**: Non-contraction subcutaneous Fat tissue Echo-Intensity; **Con MT**: Contraction Muscle Thickness; **Con MEI**: Contraction Muscle Echo-Intensity; **Con FT**: Contraction subcutaneous Fat tissue Thickness; **Con FEI**: Contraction subcutaneous Fat tissue Echo-Intensity; **MT Difference**: Difference between contraction and non-contraction Muscle Thickness; **MEI Difference**: Difference between contraction and non-contraction Muscle Echo-Intensity; **FT Difference**: Difference between contraction and non-contraction subcutaneous Fat tissue Thickness; **FEI Difference**: Difference between contraction and non-contraction subcutaneous Fat tissue Echo-Intensity.  ***p<0.05; **p<0.001** | | | | | | | | | |

**Supplementary Appendix G.** Confirmatory factor analysis and exploratory hierarchical cluster

**Correlation Matrix ^a^**

| **Correlation** | **Non-con MEI** | **Non-con MT** | **Non-con FEI** | **Non-con FT** | **Con MEI** | **Con MT** | **Con FEI** | **Con FT** |
| --- | --- | --- | --- | --- | --- | --- | --- | --- |
| **Non-con MEI**  **Non-con MT**  **Non-con FEI**  **Non-con FT**  **Con MEI**  **Con MT**  **Con FEI**  **Con FT** | 1,000 | -,161 | -,278 | -,788 | ,786 | -,111 | -,421 | -,764 |
|  | -,161 | 1,000 | -,525 | ,084 | -,260 | ,867 | -,455 | ,073 |
|  | -,278 | -,525 | 1,000 | ,588 | -,310 | -,477 | ,856 | ,596 |
|  | -,788 | ,084 | ,588 | 1,000 | -,736 | ,050 | ,641 | ,978 |
|  | ,786 | -,260 | -,310 | -,736 | 1,000 | -,220 | -,270 | -,757 |
|  | -,111 | ,867 | -,477 | ,050 | -,220 | 1,000 | -,510 | ,043 |
|  | -,421 | -,455 | ,856 | ,641 | -,270 | -,510 | 1,000 | ,667 |
|  | -,764 | ,073 | ,596 | ,978 | -,757 | ,043 | ,667 | 1,000 |

aDeterminant = 2,819E-005

| **Kaiser-Meyer-Olkin and Bartlett's Test of Sphericity** | | |
| --- | --- | --- |
| Kaiser-Meyer-Olkin Measure of Sampling Adequacy. | | ,548 |
| Bartlett's Test of Sphericity | Approx. Chi-Square | 686,205 |
|  | df | 28 |
|  | Sig. | ,000 |

| **Communalities** | | |
| --- | --- | --- |
|  | Baseline | Extraction |
| **Non-con MEI** | 1,000 | ,769 |
| **Non-con MT** | 1,000 | ,872 |
| **Non-con FEI** | 1,000 | ,824 |
| **Non-con FT** | 1,000 | ,935 |
| **Con MEI** | 1,000 | ,780 |
| **Con MT** | 1,000 | ,845 |
| **Con FEI** | 1,000 | ,860 |
| **Con FT** | 1,000 | ,942 |
| Extraction Method: Principal Component Analysis | | |

| **Total Explained Variance** | | | | | | | | | | |
| --- | --- | --- | --- | --- | --- | --- | --- | --- | --- | --- |
| **Factor** | **Initial Eigenvalues** | | | **Extraction Sums of Squared Loadings** | | | **Sum of the rotation squared saturations** | | |  |
|  | **Total** | **%**  **Variance** | **%**  **Accumulated** | **Total** | **%**  **Variance** | **% Accumulated** | **Total** | **%**  **Variance** | **% Accumulated** |  |
| 1 | 4,220 | 52,745 | 52,745 | 4,220 | 52,745 | 52,745 | 4,018 | 50,220 | 50,220 |  |
| 2 | 2,609 | 32,607 | 85,352 | 2,609 | 32,607 | 85,352 | 2,811 | 35,132 | 85,352 |  |
| 3 | ,513 | 6,413 | 91,765 |  |  |  |  |  |  |  |
| 4 | ,284 | 3,553 | 95,318 |  |  |  |  |  |  |  |
| 5 | ,153 | 1,914 | 97,232 |  |  |  |  |  |  |  |
| 6 | ,145 | 1,812 | 99,044 |  |  |  |  |  |  |  |
| 7 | ,064 | ,802 | 99,846 |  |  |  |  |  |  |  |
| 8 | ,012 | ,154 | 100,000 |  |  |  |  |  |  |  |
| Extraction Method: Principal Component Analysis, which *detected one factor formed by four ultrasound*  *outcomes with Eigenvalues above 1, explaining 52.75% of the total variance.* | | | | | | | | | | |

| **Factor Matrix ^a^** | | |
| --- | --- | --- |
|  | **Factor** | |
|  | 1 | 2 |
| **Non-con MEI** | -,795 | -,370 |
| **Non-con MT** | -,133 | ,924 |
| **Non-con FEI** | ,747 | -,516 |
| **Non-con FT** | ,943 | ,215 |
| **Con MEI** | -,750 | -,467 |
| **Con MT** | -,167 | ,904 |
| **Con FEI** | ,795 | -,478 |
| **Con FT** | ,949 | ,202 |
| Extraction Method: Principal Component Analysis | | |
| a. 2 extracted factors | | |

| **Rotated Factor Matrix ^a^** | | |
| --- | --- | --- |
|  | **Factor** | |
|  | 1 | 2 |
| **Non-con MEI** | -,875 |  |
| **Non-con MT** | ,203 | -,911 |
| **Non-con FEI** | ,515 | ,747 |
| **Non-con FT** | ,958 | ,133 |
| **Con MEI** | -,866 | ,171 |
| **Con MT** | ,164 | -,905 |
| **Con FEI** | ,574 | ,729 |
| **Con FT** | ,959 | ,147 |
| Extraction Method: Principal Component Analysis  Rotation Method: Varimax normalization with Kaiser | | |
| a The rotation converged in 3 iterations | | |

| **Factor Transformation Matrix** | | |
| --- | --- | --- |
| Factor | 1 | 2 |
| 1 | ,935 | ,354 |
| 2 | ,354 | -,935 |
| Extraction Method: Principal Component Analysis  Rotation Method: Varimax normalization with Kaiser | | |


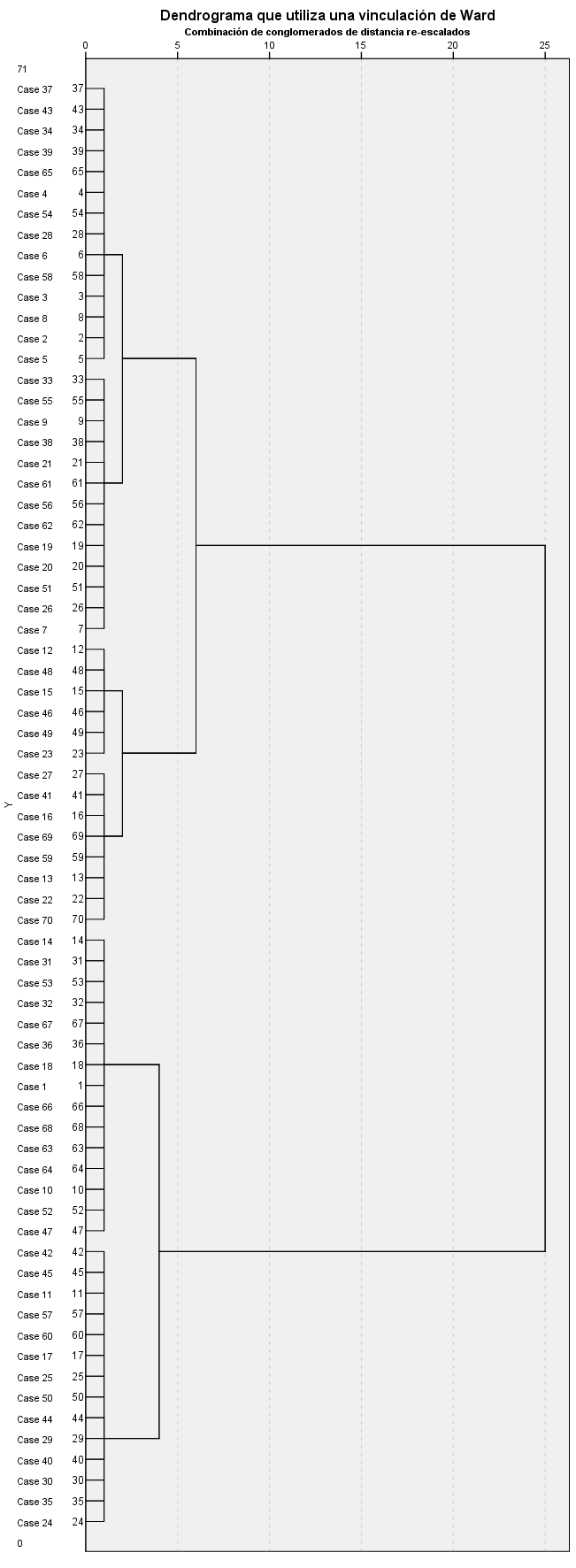


**Supplementary Appendix H.** One-way analysis of variance (ANOVA) in all outcomes among the four ultrasound groups.

| **Test of homogeneity of variances** | | | | |
| --- | --- | --- | --- | --- |
|  | **Levene's test** | **gl1** | **gl2** | **p** |
| **Non-con MT** | 3,315 | 3 | 66 | ,025 |
| **Non-con FT** | 4,175 | 3 | 66 | ,009 |
| **Non-con MEI** | 2,637 | 3 | 66 | ,057 |
| **Non-con FEI** | 1,152 | 3 | 66 | ,335 |
| **Con MT** | 1,273 | 3 | 66 | ,291 |
| **Con FT** | 5,409 | 3 | 66 | ,002 |
| **Con MEI** | 5,273 | 3 | 66 | ,003 |
| **Con FEI** | 3,038 | 3 | 66 | ,035 |

| **One-way analysis of variance (ANOVA)** | | | | | | |
| --- | --- | --- | --- | --- | --- | --- |
|  | | **Square Sum** | **gl** | **Root Mean Square** | **F** | **p** |
| **Non-con MT** | **Inter-groups** | 2,757 | 3 | ,919 | 3,412 | ,022 |
|  | **Intra-groups** | 17,777 | 66 | ,269 |  |  |
|  | **Total** | 20,533 | 69 |  |  |  |
| **Non-con FT** | **Inter-groups** | 28,112 | 3 | 9,371 | 170,829 | ,000 |
|  | **Intra-groups** | 3,620 | 66 | ,055 |  |  |
|  | **Total** | 31,732 | 69 |  |  |  |
| **Non-con MEI** | **Inter-groups** | 136426,574 | 3 | 45475,525 | 68,083 | ,000 |
|  | **Intra-groups** | 41412,351 | 66 | 667,941 |  |  |
|  | **Total** | 177838,925 | 69 |  |  |  |
| **Non-con FEI** | **Inter-groups** | 20739,914 | 3 | 6913,305 | 16,074 | ,000 |
|  | **Intra-groups** | 28386,152 | 66 | 430,093 |  |  |
|  | **Total** | 49126,066 | 69 |  |  |  |
| **Con MT** | **Inter-groups** | 2,862 | 3 | ,954 | 3,197 | ,029 |
|  | **Intra-groups** | 19,694 | 66 | ,298 |  |  |
|  | **Total** | 22,556 | 69 |  |  |  |
| **Con FT** | **Inter-groups** | 25,250 | 3 | 8,417 | 148,568 | ,000 |
|  | **Intra-groups** | 3,739 | 66 | ,057 |  |  |
|  | **Total** | 28,990 | 69 |  |  |  |
| **Con MEI** | **Inter-groups** | 101480,052 | 3 | 33826,684 | 60,360 | ,000 |
|  | **Intra-groups** | 33624,975 | 66 | 560,416 |  |  |
|  | **Total** | 135105,027 | 69 |  |  |  |
| **Con FEI** | **Inter-groups** | 17454,796 | 3 | 5818,265 | 17,462 | ,000 |
|  | **Intra-groups** | 21991,082 | 66 | 333,198 |  |  |
|  | **Total** | 39445,878 | 69 |  |  |  |

**Ultrasound outcomes differences in men between the four ultrasound groups**

| **Test of homogeneity of variances** | | | | |
| --- | --- | --- | --- | --- |
|  | **Levene’s test** | **gl1** | **gl2** | **P** |
| **Non-con MT** | ,857 | 3 | 26 | ,476 |
| **Non-con MEI** | ,173 | 3 | 26 | ,914 |
| **Non-con FT** | 13,703 | 3 | 26 | ,000 |
| **Non-con FEI** | 3,210 | 3 | 26 | ,039 |
| **Con MT** | 1,085 | 3 | 26 | ,373 |
| **Con MEI** | 1,931 | 3 | 26 | ,152 |
| **Con FT** | 10,488 | 3 | 26 | ,000 |
| **Con FEI** | 3,843 | 3 | 26 | ,021 |

| **One-way analysis of variance (ANOVA)** | | | | | | |
| --- | --- | --- | --- | --- | --- | --- |
|  | | **Square Sum** | **gl** | **Root Mean Square** | **F** | **p** |
| **Non-con MT** | **Inter-groups** | 1,394 | 3 | ,465 | 1,470 | ,246 |
|  | **Intra-groups** | 8,218 | 26 | ,316 |  |  |
|  | **Total** | 9,612 | 29 | 15579,748 | 24,505 |  |
| **Non-con MEI** | **Inter-groups** | 46739,245 | 3 | 635,766 |  | ,000 |
|  | **Intra-groups** | 15258,393 | 26 |  |  |  |
|  | **Total** | 61997,638 | 29 |  |  |  |
| **Non-con FT** | **Inter-groups** | 8,566 | 3 | 2,855 | 42,202 | ,000 |
|  | **Intra-groups** | 1,759 | 26 | ,068 |  |  |
|  | **Total** | 10,325 | 29 |  |  |  |
| **Non-con FEI** | **Inter-groups** | 6704,028 | 3 | 2234,676 | 5,211 | ,006 |
|  | **Intra-groups** | 11149,835 | 26 | 428,840 |  |  |
|  | **Total** | 17853,863 | 29 |  |  |  |
| **Con MT** | **Inter-groups** | 1,158 | 3 | ,386 | ,914 | ,448 |
|  | **Intra-groups** | 10,979 | 26 | ,422 |  |  |
|  | **Total** | 12,137 | 29 | 12103,431 | 12,096 |  |
| **Con MEI** | **Inter-groups** | 26432,721 | 3 | 1000,608 |  | ,000 |
|  | **Intra-groups** | 20924,175 | 26 |  |  |  |
|  | **Total** | 47356,895 | 29 |  |  |  |
| **Con FT** | **Inter-groups** | 6,880 | 3 | 2,293 | 28,880 | ,000 |
|  | **Intra-groups** | 2,065 | 26 | ,079 |  |  |
|  | **Total** | 8,945 | 29 |  |  |  |
| **Con FEI** | **Inter-groups** | 6500,539 | 3 | 2166,846 | 6,163 | ,003 |
|  | **Intra-groups** | 9141,167 | 26 | 351,583 |  |  |
|  | **Total** | 15641,706 | 29 |  |  |  |

**Ultrasound outcomes differences in women between the four ultrasound groups**

| **Test of homogeneity of variances** | | | | |
| --- | --- | --- | --- | --- |
|  | **Levene’s test** | **gl1** | **gl2** | **p** |
| **Non-con MT** | 2,175 | 3 | 36 | ,108 |
| **Non-con FT** | 2,867 | 3 | 36 | ,050 |
| **Non-con MEI** | 2,287 | 3 | 36 | ,096 |
| **Non-con FEI** | 1,583 | 3 | 36 | ,210 |
| **Con MT** | ,562 | 3 | 36 | ,643 |
| **Con FT** | 2,765 | 3 | 36 | ,056 |
| **Con MEI** | 1,204 | 3 | 36 | ,324 |
| **Con FEI** | ,698 | 3 | 36 | ,559 |

| **One-way analysis of variance (ANOVA)** | | | | | | |
| --- | --- | --- | --- | --- | --- | --- |
|  | | **Square Sum** | **gl** | **Root Mean Square** | **F** | **p** |
| **Non-con MT** | **Inter-groups** | 1,138 | 3 | ,379 | 1,434 | ,249 |
|  | **Intra-groups** | 9,522 | 36 | ,265 |  |  |
|  | **Total** | 10,660 | 39 |  |  |  |
| **Non-con FT** | **Inter-groups** | 15,510 | 3 | 5,170 | 112,623 | ,000 |
|  | **Intra-groups** | 1,653 | 36 | ,046 |  |  |
|  | **Total** | 17,163 | 39 |  |  |  |
| **Non-con MEI** | **Inter-groups** | 70630,799 | 3 | 23543,600 | 51,394 | ,000 |
|  | **Intra-groups** | 15575,459 | 36 | 458,102 |  |  |
|  | **Total** | 86206,257 | 39 |  |  |  |
| **Non-con FEI** | **Inter-groups** | 10877,327 | 3 | 3625,776 | 7,937 | ,000 |
|  | **Intra-groups** | 16446,514 | 36 | 456,848 |  |  |
|  | **Total** | 27323,840 | 39 |  |  |  |
| **Con MT** | **Inter-groups** | 1,143 | 3 | ,381 | 1,675 | ,190 |
|  | **Intra-groups** | 8,186 | 36 | ,227 |  |  |
|  | **Total** | 9,329 | 39 |  |  |  |
| **Con FT** | **Inter-groups** | 14,640 | 3 | 4,880 | 112,655 | ,000 |
|  | **Intra-groups** | 1,559 | 36 | ,043 |  |  |
|  | **Total** | 16,200 | 39 |  |  |  |
| **Con MEI** | **Inter-groups** | 60758,423 | 3 | 20252,808 | 70,918 | ,000 |
|  | **Intra-groups** | 9138,551 | 36 | 285,580 |  |  |
|  | **Total** | 69896,974 | 39 |  |  |  |
| **Con FEI** | **Inter-groups** | 7083,409 | 3 | 2361,136 | 7,159 | ,001 |
|  | **Intra-groups** | 11873,696 | 36 | 329,825 |  |  |
|  | **Total** | 18957,105 | 39 |  |  |  |

**Physical functional performance differences in all subjects between the four ultrasound groups**

| **Test of homogeneity of variances** | | | | |
| --- | --- | --- | --- | --- |
|  | **Levene’s test** | **gl1** | **gl2** | **p** |
| **AGE** | ,305 | 3 | 66 | ,822 |
| **HEIGHT** | 1,087 | 3 | 66 | ,361 |
| **WEIGHT** | 3,212 | 3 | 66 | ,028 |
| **BMI** | 6,026 | 3 | 66 | ,001 |
| **5-STS** | 1,545 | 3 | 66 | ,211 |
| **SPPB** | ,387 | 3 | 66 | ,762 |
| **TUG** | 3,836 | 3 | 66 | ,014 |
| **6MWT** | ,155 | 3 | 66 | ,926 |
| **UGS** | ,222 | 3 | 66 | ,881 |
| **FGS** | ,308 | 3 | 66 | ,819 |
| **KATZ INDEX** | 2,220 | 3 | 66 | ,094 |
| **LAWTON & BRODY** | 1,989 | 3 | 66 | ,124 |

| **One-way analysis of variance (ANOVA)** | | | | | | |
| --- | --- | --- | --- | --- | --- | --- |
|  | | **Square Sum** | **gl** | **Root Mean Square** | **F** | **p** |
| **AGE** | **Inter-groups** | 67,129 | 3 | 22,376 | ,618 | ,606 |
|  | **Intra-groups** | 2390,871 | 66 | 36,225 |  |  |
|  | **Total** | 2458,000 | 69 |  |  |  |
| **HEIGHT** | **Inter-groups** | 462,088 | 3 | 154,029 | 2,358 | ,080 |
|  | **Intra-groups** | 4310,998 | 66 | 65,318 |  |  |
|  | **Total** | 4773,086 | 69 |  |  |  |
| **WEIGHT** | **Inter-groups** | 1678,314 | 3 | 559,438 | 3,016 | ,036 |
|  | **Intra-groups** | 12241,080 | 66 | 185,471 |  |  |
|  | **Total** | 13919,394 | 69 |  |  |  |
| **BMI** | **Inter-groups** | 452,737 | 3 | 150,912 | 5,365 | ,002 |
|  | **Intra-groups** | 1856,385 | 66 | 28,127 |  |  |
|  | **Total** | 2309,121 | 69 |  |  |  |
| **5-STS** | **Inter-groups** | 153,373 | 3 | 51,124 | 1,770 | ,161 |
|  | **Intra-groups** | 1906,554 | 66 | 28,887 |  |  |
|  | **Total** | 2059,928 | 69 |  |  |  |
| **SPPB** | **Inter-groups** | 62,408 | 3 | 20,803 | 3,373 | ,024 |
|  | **Intra-groups** | 407,077 | 66 | 6,168 |  |  |
|  | **Total** | 469,486 | 69 |  |  |  |
| **TUG** | **Inter-groups** | 586,762 | 3 | 195,587 | 3,523 | ,020 |
|  | **Intra-groups** | 3664,489 | 66 | 55,523 |  |  |
|  | **Total** | 4251,251 | 69 |  |  |  |
| **6MWT** | **Inter-groups** | 52629,894 | 3 | 17543,298 | 1,990 | ,124 |
|  | **Intra-groups** | 581698,677 | 66 | 8813,616 |  |  |
|  | **Total** | 634328,571 | 69 |  |  |  |
| **UGS** | **Inter-groups** | ,318 | 3 | ,106 | 2,568 | ,062 |
|  | **Intra-groups** | 2,724 | 66 | ,041 |  |  |
|  | **Total** | 3,042 | 69 |  |  |  |
| **FGS** | **Inter-groups** | ,367 | 3 | ,122 | 1,699 | ,176 |
|  | **Intra-groups** | 4,756 | 66 | ,072 |  |  |
|  | **Total** | 5,123 | 69 |  |  |  |
| **KATZ INDEX** | **Inter-groups** | 1,607 | 3 | ,536 | ,608 | ,612 |
|  | **Intra-groups** | 58,165 | 66 | ,881 |  |  |
|  | **Total** | 59,771 | 69 |  |  |  |
| **LAWTON & BRODY** | **Inter-groups** | 1,861 | 3 | ,620 | ,263 | ,852 |
|  | **Intra-groups** | 155,911 | 66 | 2,362 |  |  |
|  | **Total** | 157,771 | 69 |  |  |  |

**Physical functional performance differences in men between the four ultrasound groups**

| **Test of homogeneity of variances** | | | | |
| --- | --- | --- | --- | --- |
|  | **Levene’s test** | **gl1** | **gl2** | **P** |
| **AGE** | 1,050 | 3 | 26 | ,387 |
| **HEIGHT** | ,780 | 3 | 26 | ,516 |
| **WEIGHT** | 1,872 | 3 | 26 | ,159 |
| **BMI** | 1,257 | 3 | 26 | ,310 |
| **5-STS** | 1,322 | 3 | 26 | ,289 |
| **SPPB** | 2,471 | 3 | 26 | ,084 |
| **TUG** | ,786 | 3 | 26 | ,513 |
| **6MWT** | 1,832 | 3 | 26 | ,166 |
| **UGS** | 1,864 | 3 | 26 | ,160 |
| **FGS** | 1,005 | 3 | 26 | ,406 |
| **KATZ INDEX** | 3,054 | 3 | 26 | ,046 |
| **LAWTON & BRODY** | ,553 | 3 | 26 | ,651 |

| **One-way analysis of variance (ANOVA)** | | | | | | |
| --- | --- | --- | --- | --- | --- | --- |
|  | | **Square Sum** | **gl** | **Root Mean Square** | **F** | **p** |
| **AGE** | **Inter-groups** | 290,440 | 3 | 96,813 | 2,308 | ,100 |
|  | **Intra-groups** | 1090,526 | 26 | 41,943 |  |  |
|  | **Total** | 1380,967 | 29 |  |  |  |
| **HEIGHT** | **Inter-groups** | 100,735 | 3 | 33,578 | ,971 | ,421 |
|  | **Intra-groups** | 899,132 | 26 | 34,582 |  |  |
|  | **Total** | 999,867 | 29 |  |  |  |
| **WEIGHT** | **Inter-groups** | 657,174 | 3 | 219,058 | 1,605 | ,212 |
|  | **Intra-groups** | 3549,274 | 26 | 136,511 |  |  |
|  | **Total** | 4206,448 | 29 |  |  |  |
| **BMI** | **Inter-groups** | 114,713 | 3 | 38,238 | 2,063 | ,130 |
|  | **Intra-groups** | 481,884 | 26 | 18,534 |  |  |
|  | **Total** | 596,597 | 29 |  |  |  |
| **5-STS** | **Inter-groups** | 95,928 | 3 | 31,976 | 1,456 | ,250 |
|  | **Intra-groups** | 571,088 | 26 | 21,965 |  |  |
|  | **Total** | 667,016 | 29 |  |  |  |
| **SPPB** | **Inter-groups** | 48,112 | 3 | 16,037 | 2,663 | ,069 |
|  | **Intra-groups** | 156,588 | 26 | 6,023 |  |  |
|  | **Total** | 204,700 | 29 |  |  |  |
| **TUG** | **Inter-groups** | 59,690 | 3 | 19,897 | ,485 | ,696 |
|  | **Intra-groups** | 1066,302 | 26 | 41,012 |  |  |
|  | **Total** | 1125,993 | 29 |  |  |  |
| **6MWT** | **Inter-groups** | 11850,482 | 3 | 3950,161 | ,338 | ,798 |
|  | **Intra-groups** | 303473,684 | 26 | 11672,065 |  |  |
|  | **Total** | 315324,167 | 29 |  |  |  |
| **UGS** | **Inter-groups** | ,120 | 3 | ,040 | ,683 | ,570 |
|  | **Intra-groups** | 1,524 | 26 | ,059 |  |  |
|  | **Total** | 1,644 | 29 |  |  |  |
| **FGS** | **Inter-groups** | ,056 | 3 | ,019 | ,181 | ,909 |
|  | **Intra-groups** | 2,700 | 26 | ,104 |  |  |
|  | **Total** | 2,757 | 29 |  |  |  |
| **KATZ INDEX** | **Inter-groups** | 1,542 | 3 | ,514 | ,674 | ,576 |
|  | **Intra-groups** | 19,825 | 26 | ,762 |  |  |
|  | **Total** | 21,367 | 29 |  |  |  |
| **LAWTON & BRODY** | **Inter-groups** | 3,474 | 3 | 1,158 | ,550 | ,652 |
|  | Intra-groups | 54,693 | 26 | 2,104 |  |  |
|  | Total | 58,167 | 29 |  |  |  |

**Physical functional performance differences in women between the four ultrasound groups**

| **Test of homogeneity of variances** | | | | |
| --- | --- | --- | --- | --- |
|  | **Levene’s test** | **gl1** | **gl2** | **p** |
| **AGE** | ,163 | 3 | 36 | ,921 |
| **HEIGHT** | ,780 | 3 | 36 | ,513 |
| **WEIGHT** | 4,996 | 3 | 36 | ,005 |
| **BMI** | 5,158 | 3 | 36 | ,005 |
| **5-STS** | 1,594 | 3 | 36 | ,208 |
| **SPPB** | ,306 | 3 | 36 | ,821 |
| **TUG** | 4,328 | 3 | 36 | ,011 |
| **6MWT** | ,436 | 3 | 36 | ,729 |
| **UGS** | ,260 | 3 | 36 | ,854 |
| **FGS** | ,139 | 3 | 36 | ,936 |
| **KATZ INDEX** | 2,567 | 3 | 36 | ,070 |
| **LAWTON & BRODY** | ,333 | 3 | 36 | ,801 |

| **One-way analysis of variance (ANOVA)** | | | | | | |
| --- | --- | --- | --- | --- | --- | --- |
|  | | **Square Sum** | **gl** | **Root Mean Square** | **F** | **p** |
| **AGE** | **Inter-groups** | 88,649 | 3 | 29,550 | 1,133 | ,349 |
|  | **Intra-groups** | 939,326 | 36 | 26,092 |  |  |
|  | **Total** | 1027,975 | 39 |  |  |  |
| **HEIGHT** | **Inter-groups** | 118,200 | 3 | 39,400 | 1,354 | ,272 |
|  | **Intra-groups** | 1047,400 | 36 | 29,094 |  |  |
|  | **Total** | 1165,600 | 39 |  |  |  |
| **WEIGHT** | **Inter-groups** | 2053,896 | 3 | 684,632 | 3,873 | ,017 |
|  | **Intra-groups** | 6364,488 | 36 | 176,791 |  |  |
|  | **Total** | 8418,384 | 39 |  |  |  |
| **BMI** | **Inter-groups** | 370,642 | 3 | 123,547 | 3,371 | ,029 |
|  | **Intra-groups** | 1319,263 | 36 | 36,646 |  |  |
|  | **Total** | 1689,905 | 39 |  |  |  |
| **5-STS** | **Inter-groups** | 237,216 | 3 | 79,072 | 2,657 | ,063 |
|  | **Intra-groups** | 1071,221 | 36 | 29,756 |  |  |
|  | **Total** | 1308,438 | 39 |  |  |  |
| **SPPB** | **Inter-groups** | 45,323 | 3 | 15,108 | 3,229 | ,034 |
|  | **Intra-groups** | 168,452 | 36 | 4,679 |  |  |
|  | **Total** | 213,775 | 39 |  |  |  |
| **TUG** | **Inter-groups** | 538,515 | 3 | 179,505 | 3,407 | ,028 |
|  | **Intra-groups** | 1896,571 | 36 | 52,683 |  |  |
|  | **Total** | 2435,086 | 39 |  |  |  |
| **6MWT** | **Inter-groups** | 31812,443 | 3 | 10604,148 | 1,772 | ,170 |
|  | **Intra-groups** | 215411,932 | 36 | 5983,665 |  |  |
|  | **Total** | 247224,375 | 39 |  |  |  |
| **UGS** | **Inter-groups** | ,184 | 3 | ,061 | 4,462 | ,009 |
|  | **Intra-groups** | ,494 | 36 | ,014 |  |  |
|  | **Total** | ,677 | 39 |  |  |  |
| **FGS** | **Inter-groups** | ,268 | 3 | ,089 | 2,374 | ,086 |
|  | **Intra-groups** | 1,356 | 36 | ,038 |  |  |
|  | **Total** | 1,624 | 39 |  |  |  |
| **KATZ INDEX** | **Inter-groups** | 1,306 | 3 | ,435 | ,442 | ,724 |
|  | **Intra-groups** | 35,469 | 36 | ,985 |  |  |
|  | **Total** | 36,775 | 39 |  |  |  |
| **LAWTON & BRODY** | **Inter-groups** | 7,024 | 3 | 2,341 | ,917 | ,443 |
|  | **Intra-groups** | 91,951 | 36 | 2,554 |  |  |
|  | **Total** | 98,975 | 39 |  |  |  |
